# The Central Role of Oxidative Stress in Diabetic Retinopathy: Advances in Pathogenesis, Diagnosis, and Therapy

**DOI:** 10.3390/diagnostics16030392

**Published:** 2026-01-26

**Authors:** Nicolas Tuli, Harry Moroz, Armaan Jaffer, Merve Kulbay, Stuti M. Tanya, Feyza Sule Aslan, Derman Ozdemir, Shigufa Kahn Ali, Cynthia X. Qian

**Affiliations:** 1 Faculty of Medicine and Health Sciences, McGill University, Montreal, QC H3G 2M1, Canada; nicolas.tuli@mail.mcgill.ca (N.T.);; 2 Faculty of Medicine, Queen’s University, Kingston, ON K7L 3N6, Canada; 3 Department of Ophthalmology & Visual Sciences, McGill University, Montreal, QC H4A 3S5, Canada; 4 Centre de Recherche de l’Hôpital Maisonneuve-Rosemont, Université de Montréal, Montreal, QC H1T 2M4, Canada; 5 Faculty of Medicine, Marmara University, 34722 Istanbul, Turkey; hbfeyzaslan@gmail.com; 6 Internal Medicine/Critical Care, Eastern New Mexico Medical Center, Roswell, NM 88201, USA; 7 Department of Ophthalmology, Centre Universitaire d’Ophtalmologie (CUO), Hôpital Maisonneuve-Rosemont, Université de Montréal, Montreal, QC H1T 2M4, Canada

**Keywords:** diabetic retinopathy, diabetic macular edema, oxidative stress, neovascularization, mitochondrial dysfunction

## Abstract

Diabetic retinopathy (DR) remains the leading cause of preventable blindness among working-age adults worldwide, driven by the growing prevalence of diabetes mellitus. The aim of this comprehensive literature review is to provide an insightful analysis of recent advances in the pathogenesis of DR, followed by a summary of emerging technologies for its diagnosis and treatment. Recent studies have explored the roles of cell death pathways, immune activation, and lipid peroxidation in the pathology of DR. However, at the core of DR pathology lies neovascularization driven by vascular endothelial growth factor (VEGF), and mitochondrial damage due to dysregulated oxidative stress. These dysregulated pathways manifest clinically as DR, with specific subtypes including non-proliferative DR, proliferative DR and diabetic macular edema, which can be diagnosed through various imaging modalities. Recently, novel advances have been made using liquid biopsy and artificial (AI)-based algorithms with the goal of transforming DR diagnostics. AI models show distinct promise with the capacity to provide automated interpretation of retinal imaging. Furthermore, conventional anti-VEGF injectable agents have revolutionized DR treatment in the past decades. Today, as the pathogenesis of DR becomes better understood, new pathways, such as the ROS-VEGF loop, are being elucidated in greater depth, enabling the development of targeted therapies. In addition, new innovations such as intravitreal implants are transforming the delivery of DR-specific medication. This paper will discuss the current understanding of the pathogenesis of DR, which is leading to new diagnostic and therapeutic tools that will transform clinical management of DR.

## 1. Introduction

Diabetic retinopathy (DR) is one of the most common microvascular complications of diabetes mellitus (DM), representing a leading cause of vision loss among working-age adults globally. According to the International Diabetes Federation, over 500 million adults (or 10.5% of adults) currently live with diabetes, with approximately 25% of these individuals affected by DR [1]. Furthermore, the global prevalence of DM is expected to rise from 463 million in 2019 to 700 million by 2045, with the number of individuals affected projected to increase from 103 million in 2020 to 161 million over the same period [2]. Within a decade of diagnosis, up to 75% of diabetics will develop some form of DR [3,4], whereas 5% will progress to vision-threatening stages such as proliferative DR (PDR) or diabetic macular edema (DME) [3]. The rising global prevalence of diabetes underscores the urgency of addressing DR as a public health priority.

Hyperglycemia disrupts retinal homeostasis, leading to increased vascular permeability, neovascularization, neuronal disruption, and inflammation, which manifests clinically as DR [5,6]. Our understanding of the pathogenesis of DR has evolved from a purely vascular framework to a complex interplay of metabolic, inflammatory, neurodegenerative, and mitochondrial mechanisms. Hyperglycemia-induced oxidative stress causes retinal endothelial damage, blood–retina barrier (BRB) breakdown, and neovascularization, which are hallmarks of DR pathogenesis. While vascular endothelial growth factor (VEGF)-driven angiogenesis remains a core pathological pathway, increasing evidence supports the involvement of parallel mechanisms, including lipid peroxidation, apoptosis, and mitochondrial dysfunction in DR pathophysiology.

Current clinical management is anchored in intravitreal anti-VEGF therapies and pan-retinal photocoagulation (PRP), particularly for vision-threatening complications such as PDR and DME. Although these interventions have substantially improved outcomes, they are limited by the need for frequent administration, treatment resistance, and inability to address the multifactorial pathogenesis of DR. This has driven the search for novel diagnostic and therapeutic modalities that extend beyond VEGF inhibition and target earlier, upstream pathogenic events. The latest research is refining our understanding of oxidative stress in DR pathogenesis, helping uncover new therapeutic targets beyond VEGF inhibition. This paper aims to delve into our current knowledge of DR pathogenesis and discuss novel therapeutic innovations, with a focus on ongoing and new clinical trials since 2020.

## 2. Physiological Oxidative Stress Within the Retina

**Figure 1:**
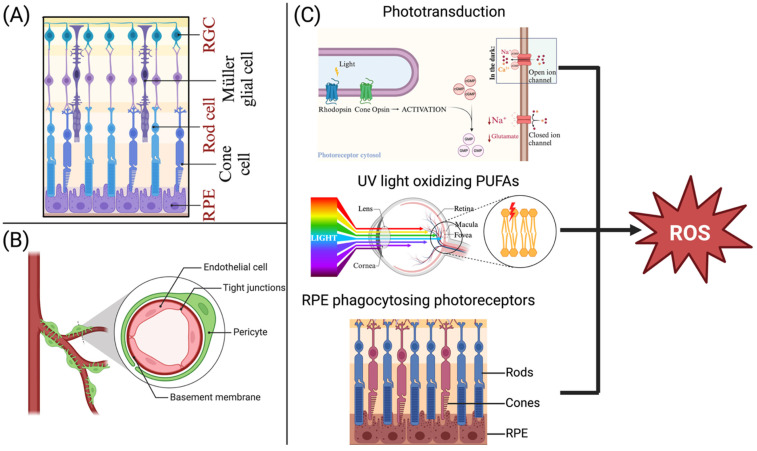
Overview of retinal physiology and homeostatic oxidative stress. (**A**) Cross-section of the retina. (**B**) Intact BRB. Pericytes surround endothelial cells, connected through tight junctions, maintaining a barrier that sequesters the retinal parenchyma from circulating proteins and cells. (**C**) Retinal oxidative stress under homeostatic conditions. Under normal conditions, the retina is exposed to constant oxidative stress due to phototransduction, oxidation of PUFAs by UV light and constant phagocytosis of photoreceptors by the RPE. Created in BioRender. Tuli, N. (2025) https://BioRender.com/p392n1o.

The retina is a highly metabolically active, multilayered neural tissue responsible for converting light into visual signals [7]. Retinal homeostasis is tightly regulated by the BRB, which comprises an inner layer (formed by tight junctions between retinal endothelial cells) and an outer layer (formed by the retinal pigment epithelium (RPE)) [8,9]. The BRB receives constant signalling from pericytes and neural and glial cells, thereby tightly regulating the retinal microenvironment to maintain immune privilege and prevent leakage of plasma proteins and toxins (Figure 1) Refs. [10,11]. Due to its high oxygen demand and high metabolic activity, the retina remains highly susceptible to oxidative damage. Even during homeostasis, photoreceptors and the RPE produce reactive oxygen species (ROS) through phototransduction, continuous phagocytosis of photoreceptors, and direct oxidation of polyunsaturated fatty acids (PUFAs) by ultraviolet light [12,13]. ROS production is usually regulated by antioxidants; however, in chronic diseases such as DR, both antioxidant impairment and ROS overproduction occur, leading to an imbalance that damages the retinal parenchyma.

In diabetes, hyperglycemia induces oxidative stress pathways in the retina, triggering a network of metabolic and inflammatory cascades. This leads to breakdown of the BRB and reduced oxygen delivery, driving neovascularization and neurodegeneration, both of which are key features of DR progression. Four main interconnected oxidative pathways are integral to DR pathogenesis: (I) advanced glycation end product (AGE) formation, (II) protein kinase C (PKC) activation, (III) the polyol pathway, and (IV) the hexosamine pathway. Persistent activation of these pathways produces ROS that drive lipid peroxidation, neovascularization, mitochondrial damage, and apoptosis, collectively amplifying oxidative stress and exacerbating retinal vascular abnormalities and neurodegeneration [14]. This section reviews the key molecular mechanisms involved in DR pathogenesis, highlighting pathways that present relevant and promising therapeutic targets.

### 2.1. Lipid Peroxidation

**Figure 2:**
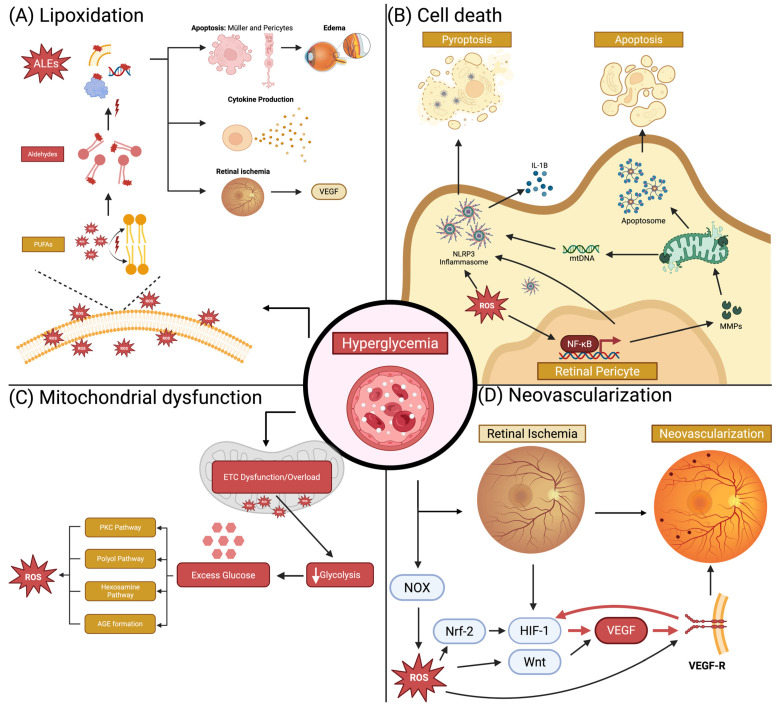
Overview of the pathogenesis of diabetic retinopathy. Hyperglycemia drives ROS production. (**A**) An Overview of ALE Production. PUFAs are oxidized by ROS, producing reactive aldehydes that react with proteins, DNA and lipids, forming ALEs. Subsequently, ALEs activate apoptosis, cytokine production and retinal ischemia. (**B**) Overview of Cell Death. ROS overload due to hyperglycemia directly triggers the NRLP3 inflammasome, leading to pyroptosis and cytokine production, causing inflammation. ROS also activate NF-κB, upregulating MMPs that will form pores in mitochondria. This will cause cytochrome c to leak out, activating the apoptosome, which triggers apoptosis. Additionally, leaked mtDNA can trigger the inflammasome. (**C**) Overview of Mitochondrial Dysfunction. Hyperglycemia-induced ETC dysfunction and overload lead to excess ROS, which impede glycolysis. As a result, excess glucose is metabolized through alternative pathways, including the PKC, polyol, and hexosamine pathways. AGEs are also formed. Altogether, these trigger ROS production. (**D**) Neovascularization. Hyperglycemia-induced hemodynamic dysfunction causes retinal ischemia, inducing HIF-1, which drives VEGF expression and neovascularization through activation of VEGF-R. Hyperglycemia also induce NOX, which produces ROS. ROS can trigger VEGF production through the VEGF autocrine loop (depicted by bolded red arrows) and through Wnt signalling. Created in BioRender. Tuli, N. (2025) https://BioRender.com/iw0cw6w.

Lipid peroxidation is a highly destructive process in which PUFAs containing carbon-carbon double bonds in the cell membrane are oxidized by ROS. The retina’s rich content of PUFAs and elevated ROS production in hyperglycemic states prime it for lipid peroxidation [15]. Lipid peroxidation begins with the oxidation of PUFAs, which form aldehydes that subsequently react with cellular macromolecules, including proteins, DNA, and other lipids, forming advanced lipoxidation end products (ALEs) (Figure 2A) [13]. Hyperglycemia-induced oxidative stress and impaired antioxidant clearance of ALEs disrupt retinal homeostasis, leading to increased ALE production. This, in turn, damages cells by impairing protein function, triggering cell death, and activating inflammatory pathways that promote angiogenesis [16,17,18,19]. Notably, in diabetic patients, levels of ALEs have been positively correlated with HbA1c, indicating that their production is associated with glycemic control [20,21].

The role of various ALEs in DR has been investigated. Studies have reported elevated ALEs, such as acrolein, 4-Hydroxy-2-Nonenal (4-HNE) and malondialdehyde in the serum, aqueous humour, and vitreous humour of diabetic patients compared to controls [13,22,23,24,25,26]. A study of MDA serum concentration in type 1 diabetics found that patients with retinopathy had higher levels ((2.65 ± 1.00) μmol/L) than those without retinopathy ((1.80 ± 0.81) μmol/L), which were higher than those of healthy controls ((1.47 ± 0.45) μmol/L) [22]. Additionally, a meta-analysis of 29 case–control studies found that patients with DR had higher levels of circulating MDA than patients without DR [25]. Notably, ALE levels may be tightly associated with glycemic control even after the onset of diabetes, with one study finding that patients with well-controlled glucose levels did not have elevated ALE levels compared with controls [27]. This was also supported by a study that found higher levels of lipid peroxides in patients with uncontrolled hyperglycemia [28]. These findings suggest that hyperglycemia is a key trigger for ALE production and its downstream effects in DR.

This link between hyperglycemia and ALE production is further supported by extensive preclinical evidence demonstrating the role of lipid peroxidation in the pathogenesis of DR. In vivo models of DR have shown that acrolein, a toxic ALE, reacts with protein nucleophiles and forms highly reactive products which trigger cytokine production, VEGF secretion, and Müller cell damage, driving the progression of DR [29,30,31]. Another ALE, 4-HNE, is also central to DR pathogenesis. It has been shown that 4-HNE can increase insulin release, promoting insulin resistance and beta cell dysfunction, thereby fortifying a hyperglycemic state [31,32,33]. Within the retina, 4-HNE reduces retinal perfusion by disrupting retinal arteriole vasodilatory responses, leading to retinal ischemia, which in turn triggers neovascularization [13,34]. It also contributes to DME development by inducing Müller cell swelling, mitochondrial dysfunction, and apoptosis [29,32]. 4-HNE also acts on retinal capillary pericytes, inducing apoptosis, leading to increased vascular permeability and subsequent edema [33]. Collectively, these findings underscore a central role of ALEs in driving both vascular and neuroglial dysfunction, thereby accelerating the progression of DR. Furthermore, clinical evidence demonstrates a correlation between glycemic control and ALEs; thus, ALEs could have biomarker potential once their precise significance is further elucidated.

### 2.2. Neovascularization

Neovascularization, defined as the formation of new blood vessels in response to retinal ischemia, is a hallmark of DR progression to the proliferative stage and causes retinal damage that jeopardizes vision [35,36]. In response to ischemia, VEGF is secreted, triggering angiogenesis to increase tissue perfusion by directly stimulating endothelial cell proliferation [37]. Members of the VEGF family include VEGF-A, VEGF-B, VEGF-C, VEGF-D, and platelet-derived growth factor, with VEGF-A being considered the most central growth factor in DR neovascularization [38,39]. Inhibition of VEGF is currently the first-line treatment for patients with PDR. However, the study of upstream and downstream pathways could yield new insights into the pathophysiology of DR and reveal new therapeutic targets.

#### 2.2.1. VEGF Production in Diabetic Retinopathy

Chronic hyperglycemia damages blood vessels and reduces oxygen-carrying capacity, triggering ischemia in organs, including the retina [40]. This hypoxic environment induces hypoxia-inducible factor 1 (HIF-1), a DNA-binding protein that binds hypoxia-responsive enhancer elements (HREs) near the VEGF gene, thereby upregulating its transcription (Figure 2D). As DR progresses, hypoxia becomes increasingly severe, causing continuous activation of the HIF-1 pathway to drive VEGF-A expression. VEGF-A binds to the VEGF receptor (VEGF-R), activating signalling cascades which form new blood vessels by inducing pericyte apoptosis, endothelial cell proliferation, and synthesis of new basement membranes for newly formed capillaries [41,42]. As hypoxia intensifies and VEGF becomes more abundant, it drives loosening of endothelial tight junctions, increasing vascular permeability, which, over time, leads to endothelial degeneration, capillary closure, and the formation of non-perfused capillaries [36]. Clinically, this manifests as vascular abnormalities, including microaneurysms, hemorrhages, cotton-wool spots, and neovascularization.

Oxidative stress is a powerful inducer of VEGF in the retina, as ROS can directly and through secondary pathways induce VEGF expression. Hyperglycemia can directly stimulate the NADPH oxidase (NOX) system, a family of membrane-associated and cytosolic proteins expressed in retinal vascular cells that use NADPH as an electron donor to drive ROS production [43,44,45]. In the retina, their activity leads to increased ROS production, which enhances VEGF expression [45]. Furthermore, excess ROS generation in retinal endothelial cells results in defective vascular repair, retinal hyperpermeability, and endothelial apoptosis, leading to microvascular damage that jeopardizes the integrity of the BRB [46,47,48,49]. Finally, NOX4 has been shown to promote neovascularization by directly stimulating the VEGF receptor through hydrogen peroxide production, amplifying VEGF potency [50]. These findings correlate with clinical studies, as a genome-wide association study found that expression of NOX-4 has been associated with more severe DR in diabetic patients [51]. Thus, these studies demonstrate the NOX system’s role in enhancing neovascularization by increasing ROS production, thereby upregulating VEGF expression and promoting microvascular damage.

Recent studies have illuminated a ROS-VEGF autocrine loop in the retina, with multiple retinal cells, including Müller cells, microglia, pericytes, and RPE cells, appearing to play integral roles in VEGF overexpression [52]. This self-sustaining loop has been demonstrated in ex vivo retinal explant models, in which induced oxidative stress or exogenous VEGF administration increases VEGF expression [53]. Mechanistically, the ROS-induced VEGF expression is driven by the redox-sensitive transcription factor Nrf2 that stabilizes HIF-1, which subsequently stimulates VEGF expression. Next, VEGF will stimulate VEGF2-R, which will further stabilize HIF-1, thereby providing positive feedback on VEGF expression [53]. As a result, there is a positive feedback loop in which ROS induces VEGF expression, which further activates its own expression through VEGF-2R, thereby amplifying neovascularization [53].

A final notable molecular pathway involved in VEGF signalling in DR is the Wnt/β-catenin pathway. The Wnt signalling pathway consists of Wnt and frizzled (Fzd) receptors that initiate an intracellular signalling cascade that results in the release of β-catenin, a transcription factor that activates specific genes [54]. In the absence of Wnt, β-catenin is bound by Axin, preventing its nuclear translocation [54]. Activated β-catenin will upregulate genes involved in several cellular functions, including proliferation, migration, tissue homeostasis, and embryogenesis [55]. Analysis of human retinas obtained from deceased patients with DR demonstrated hyperactivation of the Wnt pathway, as indicated by increased β-catenin levels [56]. This observation is supported by preclinical studies showing that hyperglycemia and excess ROS production induce Wnt signalling, leading to the expression of inflammatory cytokines and VEGF. Suppression of the Wnt signalling can down-regulate VEGF expression and ROS production, suggesting that Wnt activation in diabetic states exacerbates oxidative stress and neovascularization [54,56]. Thus, Wnt appears to regulate angiogenesis through VEGF.

#### 2.2.2. VEGF-R and Downstream Signalling Pathways

**Figure 3:**
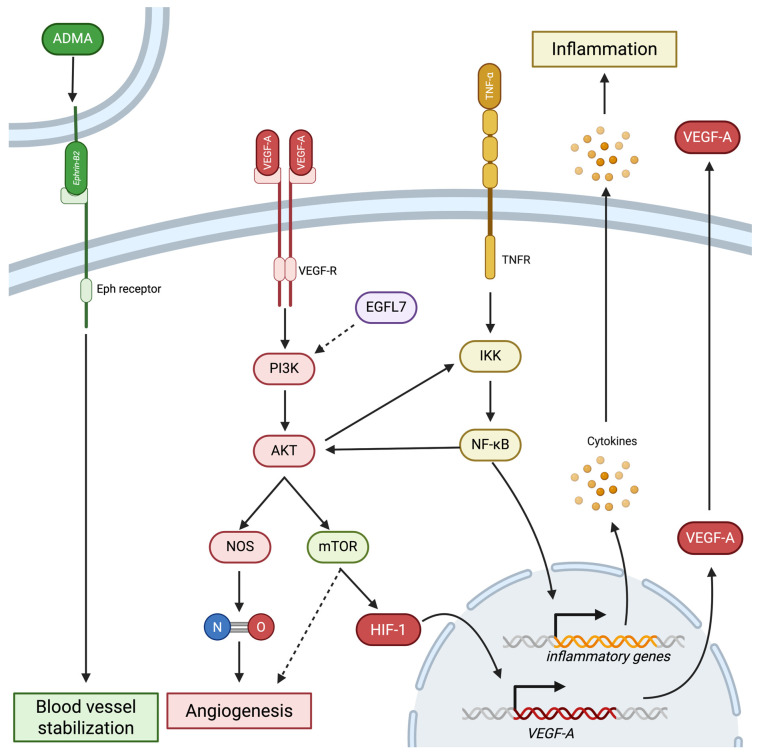
Overview of neovascularization signalling in endothelial cells. There are several pathways involved in angiogenesis. The VEGF pathway begins with VEGF-A binding to its receptors VEGF-R, causing it to dimerize and activate PI3K, which then signals to AKT, which activates two separate molecules. First, NOS produces NO, which activates angiogenesis. Second, mTOR is activated, which activates HIF-1, a transcription factor that upregulates VEGF-A expression. Notably, this pathway is regulated by EGFL7, which is upstream of PI3K. The ephrin pathway is also important in neovascularization. It begins with ADMA, which increases ephrin-2B expression that interacts with its Eph receptor, leading to blood vessel stabilization. Lastly, the NF-κB pathway interacts bidirectionally with the PI3K/AKT pathway. AKT activates IKK, thereby promoting NF-κB activation and the transcription of pro-inflammatory cytokines. In turn, NF-κB can enhance PI3K/AKT signalling, creating a positive feedback loop that amplifies inflammation and angiogenesis. (Solid arrows depict direct interactions. Dashed arrows depict multi-step pathways). Created in BioRender. Tuli, N. (2025) https://BioRender.com/7tgkhf9.

VEGF-A is the most significant activator in the retina. It acts through VEGF-R1 and VEGF-R2, which are receptor tyrosine kinases expressed by retinal vascular cells, including pericytes and endothelial cells, as well as retinal neurons, including retinal ganglion cells (RGCs), Müllers cells, and photoreceptors [36,57,58,59,60]. Upon binding to VEGF, VEGF-Rs dimerize and activate downstream signals leading to angiogenesis, increased vascular permeability, as well as cell survival and proliferation (Figure 3) [36].

The phosphoinositide 3-kinase (PI3K)/AKT pathway is a well-studied cascade downstream of VEGF-R that is vital to DR pathogenesis. Upon VEGF-R dimerization, PI3K is phosphorylated and activated, which in turn activates AKT [61]. Activation of the PI3K/AKT pathway has been shown to be induced by high glucose, hypoxia and AGEs in cultured human endothelial cells [6,61,62,63]. Upon activation, PI3K/AKT induces nitric oxide (NO) production, leading to angiogenic changes in endothelial cells [61,64]. NO production is achieved through AKT-dependent phosphorylation of the NO synthase (NOS) protein family, which then produces NO that will induce angiogenesis and increase vascular permeability [65,66,67,68]. In addition to activating NO, VEGF-R signalling has been shown to activate the transcription of growth factors involved in neovascularization, including other VEGFs, angiopoietins, and ephrins, as well as their respective receptors [69].

PI3K/AKT’s most notable downstream target is mammalian target of rapamycin (mTOR) and mTOR complexes (mTORCs), which are known to control tissue homeostasis to manage cell growth, proliferation and apoptosis [70]. mTOR activation by the VEGF-A/PI3K/AKT pathway has been shown to further induce VEGF-A expression by upregulating HIF-1 [68,71]. mTOR’s role in HIF-1-dependent VEGF-A expression is supported by the observation that inhibiting mTOR attenuates hyperglycemia-induced overexpression of VEGF-A [72]. In addition to their role in the PI3K/AKT pathway, mTORC complexes are expressed in inner retinal neurons, and their loss in DR may contribute to neurodegenerative processes observed in DR [70,73].

The regulation of the PI3K/AKT pathway is currently under investigation. Epidermal growth factor-like structural domain 7 (EFGL7) is an endothelial cell-specific angiogenic factor that is activated in response to hyperglycemia [39]. Its function is also important in regulating ube-forming processes of angiogenesis [74]. A recent study demonstrated that when EGFL7 is suppressed in endothelial cells under high-glucose conditions, the VEGF-A/PI3K/AKT pathway is downregulated, suggesting that EGFL7 is an upstream regulator of this pathway [39]. In addition, it has been shown that AKT interacts with DR-associated inflammatory mediators, specifically transcription factor NF-κB. There is evidence that AKT activates IκB kinase (IKK), which releases NF-κB to activate inflammatory genes [61,75,76]. In addition, NF-κB is activated through TNF-α signalling has been shown to induce AKT activation [77]. This dual activation pathway demonstrates the crosstalk between inflammatory and angiogenic pathways in DR.

#### 2.2.3. The Ephrin Pathway

The largest family of RTK receptors, the membrane-bound ephrin receptors (Eph), have also been shown to have an integral role in angiogenesis in DR. Eph receptors are activated by their ligands, ephrins, which are membrane-bound proteins. Notably, increased levels of ephrin-A1 and ephrin-B2 have been detected in DR patients and in animal models of DR [78,79]. However, for the angiogenic effects of the ephrin pathway to be initiated, another signal is required, and some suggest that signal may be that of VEGF [80]. Activation of the ephrin pathway normally stabilizes blood vessels by mediating communication between pericytes and endothelial cells, and is also critical for regulating angiogenic pathways activated through VEGF-signalling [78,80]. This is supported by a study in which knocking out ephrin-B2 resulted in severe vascular defects [81]. Studies have shown that blocking ephrin-B2 can inhibit pathological neovascularization in animal models, suggesting it as a potential therapeutic target for DR [82]. The exact regulation of the ephrin pathway remains to be elucidated, yet one study recently found that asymmetric dimethylarginine (ADMA), an endogenous inhibitor of nitric oxide synthase, can activate ephrin-B2 [83]. These studies present more insight into the neovascular abnormalities in DR.

### 2.3. Mitochondrial Damage

**Figure 4:**
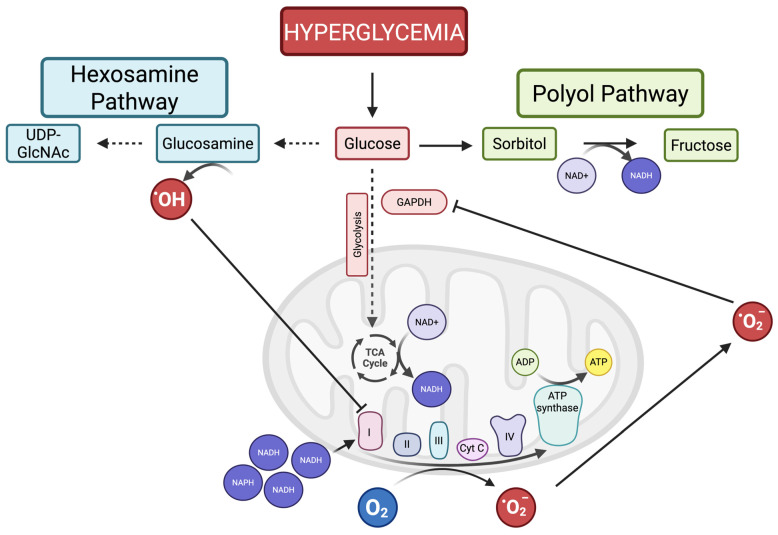
Overview of mitochondrial dysfunction in diabetic retinopathy. In a hyperglycemic state, excess superoxide (O_2_^−^) is produced due to increased ETC activity. This is partly attributed to NADH overload and NAD+ depletion. NAD+ is depleted through several pathways, including the Krebs (TCA) cycle and the polyol pathway, as sorbitol is converted to fructose while consuming NAD+. Superoxide production from ETC overload inhibits glycolysis by suppressing GAPDH, thereby diverting glucose metabolism to alternative pathways. The hexosamine pathway converts glucose to UDP-GlcNAc. Glucosamine, one of the intermediates of this pathway, produces hydrogen peroxide, which inhibits ETC function. Overall, excess glucose triggers a cascade that impairs the ETC, thereby exacerbating ROS production as excess electrons are used to convert oxygen into superoxide. (Solid depict direct interactions. Dashed arrows depict multi-step pathways. Curved, faded arrows depict associated reactions). Created in BioRender. Tuli, N. (2025) https://BioRender.com/s74na4d.

Mitochondria play a central role in DR pathogenesis, both sources and targets of oxidative stress. Under homeostatic conditions, photoreceptors and retinal neurons already have high metabolic activity, demanding significant mitochondrial activity. Under hyperglycemic conditions, retinal mitochondria become overworked and damaged. In early DR, mitochondrial function increases, followed by a decline as hyperglycemia becomes chronic, resulting in a vicious cycle of ROS production as the electron transport chain becomes overwhelmed [84,85]. The initial increase in mitochondrial activity is matched by increased ATP production; however, as hyperglycemia becomes chronic, there is an increased oxygen consumption that is not matched by ATP production due to mitochondrial uncoupling, with the excess oxygen converted to ROS [84,85,86]. Mitochondria become overloaded by excess NADH and FADH2 production through glycolysis, which in turn overwhelms the electron transport chain (ETC), resulting in an abundance of ROS (Figure 4). Over time, as oxidative stress increases, glycolysis is inhibited, leading to glucose metabolism via alternative pathways that exacerbate mitochondrial damage and oxidative stress.

#### 2.3.1. Alternate Pathways of Glucose Metabolism and NADH/NAD+ Ratio

The cellular NADH/NAD+ ratio is vital to mitochondrial function, with NAD+ depletion severely impairing mitochondrial bioenergetics. High levels of ROS, specifically superoxide, inhibit glycolysis, causing glucose to be metabolized through alternative pathways, such as the polyol, PKC, and hexosamine pathways (Figure 2C) [87]. The polyol pathway consists of oxidizing glucose to sorbitol, then to fructose. The latter reaction consumes NAD+, generating NADH and depleting NAD+ stores, thereby providing positive feedback to oxidative stress while also generating excess NADH [43,87,88]. In addition, hyperglycemia drives the Krebs cycle, which also produces NADH, while consuming NAD [89]. Lastly, poly (ADP)-ribose polymerases (PARPs), a family of DNA repair proteins, may have a role in disrupting the NADH/NAD+ ratio, as their function consumes NAD+ [84]. Some studies have shown that *PARP*-deficient mice are protected against diabetes and preserve mitochondrial function [90,91]. Together, in the oxidative state, excess activation of the polyol pathway, activation of the Krebs cycle, and PARP activity induce metabolic changes that consume NAD+ while producing excess NADH, which overloads the ETC and impairs mitochondrial bioenergetics.

NADH donates its electrons to complex I of the electron transport chain (ETC). During NADH overload, mitochondrial membrane potentials increase, which can cause stagnation of the ETC, leading to offload of electrons to oxygen molecules, generating superoxide, a potent ROS [87,92]. In addition, NAD+ depletion dysregulates mitochondrial protein synthesis, further impairing the ETC and exacerbating mitochondrial dysfunction [84]. Lastly, NAD+ has been shown to be essential for photoreceptor function, and NAD+ deficiency can disrupt mitochondrial structure and function in photoreceptors, leading to widespread photoreceptor loss that may precede vascular anomalies in DR [93]. Thus, the imbalance in NADH/NAD+ causes (1) ETC overload and stagnation, (2) NAD+ depletion impairing mitochondrial protein synthesis, and (3) impaired photoreceptor function.

As a result of ROS-mediated glycolysis inhibition in DR, the hexosamine pathway is also upregulated. This pathway consists of multiple enzymatic reactions that convert glucose into uridine-5′-diphospho-*N*-acetylglucosamine (UDP-GlcNAc), a metabolite involved in glycosylation and protein modification [94]. In hyperglycemia, persistent activation of this pathway is attributed to excessive mitochondrial ROS production, which inhibits glyceraldehyde-3-phosphate dehydrogenase (GAPDH), an enzyme central to glycolysis [14,95,96]. Activation of the hexosamine pathway amplifies ROS production, as glucosamine, an intermediate in this pathway, increases hydrogen peroxide production, which further impairs mitochondrial oxidative phosphorylation [97,98,99]. Thus, in the context of hyperglycemia, excess ROS are generated through ETC overload, which inhibits glycolytic function and activates alternate pathways of glucose metabolism, further exacerbating mitochondrial dysfunction through NADH overload and ROS production.

#### 2.3.2. Mitochondrial Dysfunction in Photoreceptors

Photoreceptors play a key role in generating oxidative stress in DR. This is supported by clinical observations that patients with retinitis pigmentosa or photoreceptor degeneration have less severe forms of DR than patients without photoreceptor degeneration [84,100]. There are two leading theories behind ETC-derived oxidative stress in photoreceptors. The first is a disruption of normal electron flow through the ETC, with electrons accumulating at complex I and III, where they react with oxygen to yield superoxide [84,101,102]. Alternatively, in other tissues such as the heart and kidney, it has been observed that β-oxidation of fatty acids causes excess ETC activation, leading to electron leakage that reacts with oxygen, producing superoxide [103,104,105]. Nevertheless, in photoreceptors of diabetic patients, ETC dysfunction or overload causes excess ROS production.

#### 2.3.3. Epigenetic Modifications

Beyond these direct effects, hyperglycemia-dependent oxidative stress induces genetic and epigenetic changes that modify mitochondrial function. Specific patterns of mitochondrial DNA (mtNDA) methylation, an epigenetic modification that alters transcriptional activity, have been associated with DR. These epigenetic modifications in response to long-term hyperglycemia are referred to as metabolic memory [97,106]. In retinal cells from patients with DR, specific patterns of nuclear DNA methylation have been observed, leading to decreased expression of antioxidative proteins that normally suppress oxidative stress [107,108]. Furthermore, mitochondrial DNA is methylated in DR, affecting both mitochondrial transcription and replication and ultimately impairing mitochondrial antioxidant functions [87,109]. Together, these studies provide evidence that DR-specific methylation patterns impair cellular antioxidative responses.

mtDNA lacks histones, making it more susceptible to oxidative stress-induced damage. The specific unwound non-coding region, called the displacement loop (D-loop), which contains essential transcription regulatory elements critical to mtDNA replication, is highly susceptible to oxidative stress-induced damage [110,111]. Under hyperglycemic conditions, the D-loop is hypermethylated and becomes smaller, leading to mitochondrial dysfunction and impaired replication even after hyperglycemia resolves [112]. Furthermore, methylation of mtDNA impairs its own repair mechanisms, leading to mismatches that, over time, decrease mitochondrial function and subsequently trigger apoptosis [113,114]. As a result of oxidative stress-induced epigenetic modifications, mitochondrial functions are compromised in the retina, leading to the progression of DR.

Mitochondrial memory is thought to be irreversible. To clarify, the epigenetic modifications are maintained even after glycemic control. One study demonstrated that histone methylation of *Sod2*, a gene encoding a mitochondrial antioxidant protein, is maintained in rats exposed to hyperglycemia for 3 months, even after 3 months of good glycemic control [115]. Furthermore, another study demonstrated that damage to the D-loop region persisted even after diabetic rats had achieved 3 months of good glycemic control [116]. Thus, the disruptive epigenetic modifications are maintained even after glycemic control is restored, demonstrating the long-term effects, or “epigenetic memory,” that drive DR progression.

#### 2.3.4. Mitochondrial Dynamics

In addition to genetic and epigenetic alterations, mitochondrial dynamics are disrupted in DR. Mitophagy, the selective degradation of damaged mitochondria, is dysregulated in DR [117]. The exact nature of this dysregulation is still debated, with some studies arguing that mitophagy is upregulated to prevent DR progression [118,119,120], while others support that hyperglycemic conditions inhibit mitophagy (Table 1) [121,122,123]. The current consensus is that mitophagy is affected by the severity of hyperglycemia, with greater inhibition at higher glucose concentrations [87,123]. Many studies have elucidated the role of mitophagy in Müller cells under hyperglycemic conditions. It has been shown that Müller cells exhibit enhanced mitophagy in high-glucose environments [118,119]. This effect is reversible by knocking down thioredoxin-interacting protein (TXNIP), an inhibitor of mitochondrial antioxidants [119,124]. This is further supported by several other studies that demonstrated that TXNIP positively regulates mitophagy in rat Müller cells under high-glucose conditions and also triggers apoptotic pathways [125,126]. It has been proposed that this serves as a protective role early in DR, by promoting the degradation of damaged mitochondria and thereby supporting cell survival [126]. However, as DR progresses, mitophagy becomes further impaired, exacerbating mitochondrial damage and ROS elimination [127].

In addition to dysfunctional mitochondrial degradation, there are reported changes in the balance of mitochondrial fusion and fission in DR. This is due to two dualling GTPases, mitofusin 2 (MFN2), which regulates fusion, and dynamin-related protein 1 (DRP1), which regulates fission [84]. In human DR, as well as in in vitro Müller and photoreceptors grown in high-glucose, it has been shown that the balance shifts favouring mitochondrial fission, with MFN2 downregulation and DRP1 upregulation [124,128]. The changes are not reversible by lowering glucose levels, and it is thought that this shift occurs due to oxidative stress or hypermethylation of the *MFN2* promoter; however, the exact mechanism remains to be elucidated [84,127,129]. It has been shown that activation of Mfn2 by a small-molecule activator can increase fusion and, in turn, increase biogenesis of new mitochondria, possibly reversing the shift in mitochondrial dynamics [127]. Thus, regulating mitochondrial fusion and fission dynamics has shown promise in pre-clinical models of DR, offering a potentially novel therapeutic strategy.

**Table 1:** Mitophagy in diabetic retinopathy models.

Author (Year)	Model	Findings of Mitophagy	References
Devi et al. (2012)	Rat Müller Cell under high-glucose	Increased mitophagy dependent on thioredoxin-interacting protein	[124]
Devi et al. (2017)	Rat Müller Cell under high-glucose	Increased mitophagy dependent on thioredoxin-interacting protein	[119]
Huang et al. (2018)	Human RPE under high-glucose	Increased mitophagy	[120]
Kowluru et al. (2021)	Human retinal endothelial cells	Increased mitophagy	[130]
Zhou et al. (2020)	Retinal ganglion cells in streptozotocin-induced diabetic rats	Increased mitophagy reversible with liraglutide	[131]
Hombrebueno et al. (2019)	Mitophagy-reporter mouse model	Initial increase in mitophagy then mitophagy deterioration	[121]
Zhang et al. (2019)	Retinal pigment epithelium cells under high-glucose	Decreased mitophagy	[123]
Zhou et al. (2019)	Diabetic mice (db/db)	Mitophagy was decreased in diabetic mouse, but enhanced with treatment with notoginsnoside R1	[118]
Sun et al. (2021)	Müller cells, endothelial cells and photoreceptors under hypoxic conditions	Decreased mitophagy reversible with bevacizumab	[132]
Xie et al. (2021)	Human retinal capillary cells under high-glucose	Decreased mitophagy	[122]

### 2.4. Cell Death

Cell death is prominent in DR and is primarily mediated by two pathways: apoptosis and pyroptosis Apoptosis is a specific subtype of programmed cell death activated by regulated pathways, resulting in cell shrinkage and DNA fragmentation that form apoptotic bodies, which are cleared by the immune system [133,134]. Retinal apoptosis is prominent and an early defining feature of DR. Histological studies of human and rodent retinas have identified apoptosis of pericytes and endothelial cells in retinal capillaries even prior to clinical manifestations [135]. Pericytes are cells that maintain capillary integrity and are highly vulnerable to apoptosis because they do not regenerate. The excessive apoptosis of pericytes in DR leads to the formation of acellular capillaries, microaneurysms, and edematous manifestations, and thus pericytes are central to retinal vascular integrity [84,136]. Apoptosis of retinal neurons and photoreceptors is also prevalent in DR, contributing to retinal thinning and functional deficits [84,137].

The widespread apoptosis in DR, involving both vascular and neuronal cells, is predominantly mediated by the intrinsic (mitochondrial) pathway. In diabetic mouse models, NF-κB activation in retinal cells has been shown in response to oxidative stress [138]. Consequently, NF-κb positively regulates metalloproteinases (MMP) expression, a family of proteins that, when overproduced, increase mitochondrial permeability by forming pores in the mitochondrial membrane [139]. Consequently, cytochrome c leaks into the cytosol through these newly formed pores, triggering the apoptosome. The apoptosome terminates with caspase-3 activation, a protease that triggers apoptosis (Figure 2B). Hyperglycemia can also induce the release of apoptosis-inducing factor (AIF) from mitochondria, which then translocates to the nucleus to induce apoptosis through direct DNA fragmentation [140].

In addition to apoptosis, pyroptosis is a form of inflammatory cell death that is also prominent in DR. Pyroptosis is triggered by NLRP3 inflammasome activation. In DR, low-grade inflammation is prevalent, marked by elevated levels of several pro-inflammatory cytokines, including IL-1, IL-6, and TNF, detected in the aqueous and vitreous humour of patients with DR [141,142]. Furthermore, there is significant evidence that the NLRP3 inflammasome is elevated in vitreous samples from patients with DR, and its activity correlates with disease severity, linking inflammasome activation to DR pathology [143,144]. The NLRP3 inflammasome is an intracellular immune factor composed of a sensor protein, an adaptor protein, and an effector protein (caspase-1). In DR, hyperglycemia-dependent oxidative stress and inflammatory signals activate NLRP3. This activation drives disease progression by triggering pyroptosis, leading to the loss of retinal vascular cells and neurons and further amplifying retinal inflammation through the production of IL-1 and IL-18 (Figure 2B) [141].

Many in vivo and in vitro animal studies have demonstrated that hyperglycemia and excessive oxidative stress induce pyroptosis through activating NLRP3 inflammasome in virtually all retinal cells, with a focus on pericytes, retinal endothelial cells, and Müller cells [141,145,146,147,148,149]. Additionally, a systematic review found that NLRP3 inflammasome biomarkers IL-1β and IL-18 were elevated in the vitreous and serum of diabetic patients [150]. One protein that appears central to inflammasome activation in DR is connexin 43, which is ubiquitously expressed in the retina and functions as a gap junction for cell–cell communication [141,151]. In DR, connexin 43 channels have been shown to be upregulated, and their irregular opening in DR triggers the NRLP3 inflammasome, resulting in the production of inflammatory cytokines that increase vascular permeability and the activation and propagation of pyroptosis from cell to cell [151,152]. The ubiquitous induction of cell death throughout the retina due to connexin 43-mediated NLRP3 activation leads to retinal vascular breakdown, with vascular leakage and ischemia. Additionally, loss of neurons results in retinal neurodegeneration, and inflammasome-triggered inflammation drives progression of DR.

## 3. Clinical Presentation

**Figure 5:**
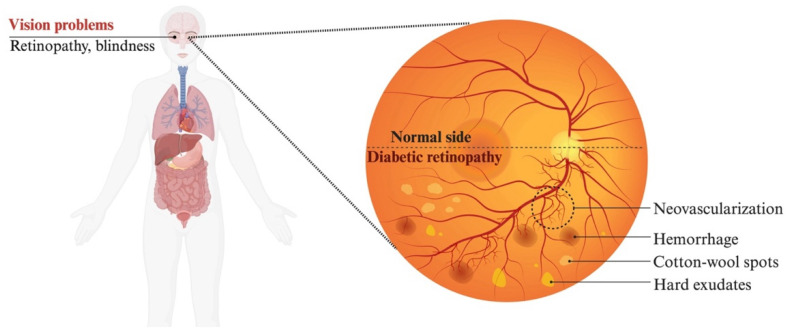
Schematic illustration of fundoscopy findings associated with diabetic retinopathy. Fundoscopy is the screening tool for DR, and can detect hemorrhage, cotton-wool spots and hard exudates, all indicative of NPDR. It can also detect neovascularization, indicative of PDR. Created in BioRender. Tuli, N. (2025) https://BioRender.com/36zwm05.

DR is marked by a subclinical progression, with patients in the early to moderate stages of non-proliferative diabetic retinopathy (NPDR) often remaining asymptomatic [153,154,155]. However, as the disease advances to severe NPDR or PDR, which can be established by funduscopic examination (Figure 5) and complementary imaging modality assessment, significant and potentially disabling vision loss can occur [153,154,155]. The onset of DME, vitreous hemorrhage, and retinal detachment may also lead to symptoms of vision impairment [153,154,155,156,157]. Symptoms, such as blurred or double vision, visual distortion, floaters, and changes in refractive error, can vary in intensity both throughout the day and from day to day [155]. PDR or DME-induced vision loss can significantly affect patients’ quality of life, including independence, psychosocial well-being, productivity, mobility, leisure, and self-care activities [154,155,158,159].

### 3.1. Ophthalmic Comorbidities Associated with Diabetic Retinopathy

Given the systemic nature of DM, several other ocular conditions, such as glaucoma, cataract, and age-related macular degeneration (AMD), have been studied as comorbidities [160].

#### 3.1.1. Glaucoma

Neovascular glaucoma (NVG) is a recognized complication associated with DM, closely linked to PDR [161,162]. DM is a key factor in its underlying etiology, with the risk further heightened by procedures such as vitrectomy and cataract surgery [160,161,162,163,164]. NVG is secondary to PDR, with excess production of angiogenic factors in response to retinal ischemia driving neovascularization in the iris as well. Subsequent fibrovascular membrane proliferations cause thickening of the iris, which narrows the anterior chamber angle and thereby blocks the trabecular meshwork. Additionally, peripheral anterior iris adhesions can further close the angle [164]. Together, these impede aqueous humour drainage, increasing intraocular pressure (IOP), triggering glaucoma.

Conflicting evidence has been reported surrounding the association between open-angle glaucoma (OAG) and DM. Large population-based studies, such as the Framingham Heart Study, the Baltimore Eye Survey, the Rotterdam Eye Study, and the Barbados Eye Study, did not establish a significant association between DM and open-angle glaucoma [160,165,166,167,168]. However, the Blue Mountains Eye Study, the Los Angeles Latino Eye Study, and a longitudinal cohort study involving more than 2 million individuals aged 40 and older in the United States found an increased risk of developing open-angle glaucoma in patients with DM. The pathophysiological link between DR and OAG is also not fully understood. It is hypothesized that similarities in their pathogenesis cause the comorbidity. Predominantly, DR causes neurodegeneration, including RGC loss and microvascular dysfunction, leading to optic nerve head ischemia, both of which are hallmarks of OAG. This is the current leading theory describing a pathophysiological link between DR and OAG [169].

#### 3.1.2. Cataracts

An association between cataracts and DM is well-documented, with diabetic patients being up to five times more likely to develop cataracts [160,170]. A meta-analysis found that patients with Type 2 DM had an increased risk of developing posterior cortical and posterior subcapsular cataracts, with the risk further increased by longer disease duration and poor glycemic control [171,172]. Furthermore, in patients with uncontrolled type 1 DM, the development of a snowflake (diabetic) cataract has been observed as a rare clinical manifestation [160,173,174]. While cataract surgery generally produces excellent outcomes, it can lead to DR progression and a fourfold increased risk of DME development in patients with DM [175].

The pathogenesis of diabetic cataracts is quite similar to that of DR. Of utmost importance, the polyol pathway is upregulated in the lenses of DM patients in response to hyperglycemia. As a result, excess sorbitol accumulates with water draws, causing fibre swelling and damage, opacifying the lens [176]. Furthermore, oxidative stress is also significant in the diabetic lens, as the polyol pathway both depletes antioxidants and generates ROS [177]. In addition, hyperglycemia also induces AGE formation in the lens, contributing to ROS production [178]. This enhanced oxidative stress damages proteins and lipids in the lens, leading to opacification [176]. Thus, diabetic cataract formation is caused by excessive oxidative stress driven by upregulation of the polyol and AGE pathways, activated by hyperglycemia.

#### 3.1.3. Age-Related Macular Degeneration

DM is an established risk factor for AMD [179]. However, the role of DR-associated retinal pathology as a risk factor for AMD remains contentious. While some large population-based studies have identified a link between DR and both neovascular and atrophic AMD, other research has suggested that DR may have a protective effect against the development of AMD [180,181,182,183,184].

AMD and DR share a common pathophysiological mechanism, providing a plausible explanation behind their complex, controversial epidemiological relationship. Akin to DR, AMD pathogenesis has a central oxidative stress component driven by ageing and light exposure [185,186]. Furthermore, cytokine accumulation, causing chronic inflammation, exacerbates both AMD and DR progression [187]. The overlapping pathogenic mechanisms lead to similar retinal changes in both diseases, most notably RPE and BRB degeneration, ischemia, and neovascularization.

## 4. Classification and Diagnosis

Diagnosis of DR does not rely solely on clinical symptoms; instead, imaging is central to DR diagnosis, with various imaging modalities and diagnostic tools outlined in (Table 2).

### 4.1. Fundus Examination

Dilated posterior segment evaluation is vital for DR screening, as it detects several abnormalities indicative of both NPDR and PDR. Fundus photography now relies on digital imaging. Standard macular fundus photography captures 30° of the posterior pole of the eye, including the macula and the optic nerve [197]. Today, wide-field and ultra-wide-field fundus photography allow visualization of 200° of the posterior pole with montage applications [198]. With these expanded imaging capabilities, fundus photography can capture a broader range of pathological changes.

### 4.2. Fluorescein Angiography

Fluorescein angiography (FA) is critical for assessing the retinal microvasculature in DR. It was first incorporated into ophthalmologic practice in 1967 and is performed by administering sodium fluorescein, a fluorescent dye, intravenously [199]. As sodium fluorescein travels in the bloodstream and reaches the eye, it will travel through the arteries and veins of the retina. Notably, the BRB prevents the diffusion of dye into the retina [190]. İn the bloodstream, 20% of fluorescein remains unbound and is excited by the application of blue light (465–490 nm), which causes it to fluoresce, emitting a yellow-green light (520–530 nm) that is captured with a fundus camera with a specialized filter [197,200]. FA is best at visualizing retinal vasculature and limited at visualizing choroidal circulation, as the choriocapillaris allows the dye to diffuse freely, and the excitation wavelength poorly penetrates deep retinal layers, including the RPE [190]. Fluorescein is rarely used alone in the diagnosis of DR; however, when combined with fundus photography, it can classify NPDR and PDR with 51.3% and 80% sensitivity, respectively. In addition, this combination has a sensitivity of 80% for NPDR classification, which decreases to 51.3% for PDR classification [201]. Thus, alone, FA is not an optimal choice for diagnosing DR, as newer imaging modalities provide superior results.

### 4.3. Optical Coherence Tomography

Optical coherence tomography (OCT) is an innovative imaging technique that provides non-invasive, histology-like, high-resolution cross-sectional images of the retina. It captures these images by splitting a broadband light, sending one beam to a reference arm and the other to the retina. The beams are reflected and detected by a specialized detector. Reflected beams recombine, creating an interference pattern, which is detected indirectly in a method called low-coherence interferometry [197]. OCT performs many single scans, termed amplitude scans (A-scans), which detect structures at a given depth [197,202]. By moving the reference mirror, backscattered tissue intensity levels can be detected at multiple depths, thereby collecting A-scans across multiple depths of the retina. These scans are combined to form B-scans, which are high-resolution images of retinal cross-sections. In terms of clinical performance, one study employing machine-learning models found that OCT alone can diagnose and classify DR with an accuracy of 76%, with sensitivity of 85% and specificity of 87%, which notably increased to an accuracy of 92%, sensitivity of 95% and specificity of 98% when used in combination with OCT angiography (OCTA) [203].

### 4.4. Optical Coherence Tomography Angiography

OCT angiography (OCTA) builds upon the low-coherence interferometric method introduced in OCT to provide an image of the retinal vasculature [204]. Precisely, OCTA uses particle motion within retinal vessels as intrinsic contrast. By taking repeated OCT-B scans at the same location, it can generate angiograms by comparing motion-related differences between these scans [202]. With multiple scans at the exact same location, structures that remain unchanged, such as blood vessels, will have correlated signals, whereas those that change, such as moving red blood cells, will have decorrelated signals [202]. These signal differences and correlations enable the reconstruction of the retinal vasculature in a minimally invasive manner. A recent meta-analysis found that OCTA could differentiate DR from diabetics without DR with a sensitivity of 88% (95% CI: 85–92%) and specificity of 88% (95% CI: 85–96%). Notably, it could differentiate between PDR and NPDR with a sensitivity of 91% (95% CI: 86% to 95%) and specificity of 91% (95% CI:86% to 96%), demonstrating that even as a non-invasive method, it can be reliably used to diagnose DR [205].

### 4.5. Indocyanine Green Angiography

Indocyanine green angiography (ICGA) functions similarly to FA, albeit using a different fluorophore and excitation wavelength. Indocyanine green is administered intravenously and is excited by an infrared light with a wavelength between 790 and 805 nm, leading to emission at 835 nm [196]. Because infrared light with longer wavelengths penetrates the RPE and choroid, it allows superior visualization of the choroidal circulation [206]. This property also limits its ability to image the retinal microvasculature precisely.

### 4.6. B-Mode Ultrasound

B-mode ultrasounds use high-frequency sound waves, emitted by a transducer onto the underlying tissue, in this case the retina, which are reflected back to the transducer with varying amplitudes, depending on the tissue [197]. Lower reflected amplitudes correspond to low-density tissue and appear black. For example, if there were an extensive vitreous hemorrhage, the blood would appear black. Higher-density tissues, like the retina, reflect sound waves, sending back high-amplitude waves that are depicted as hyperechoic.

### 4.7. Classification of Diabetic Retinopathy

In 1968, leading experts from around the world gathered to review the understanding of DR, resulting in the development of a standardized classification and staging system known as the Airlie House Classification [207,208]. Minor modifications were made following the Diabetic Retinopathy Study (DRS) and the Early Treatment Diabetic Retinopathy Study (ETDRS), culminating in the adoption of the ETDRS severity scale. This system is regarded as the gold standard in both clinical and research practice; however, its complexity creates barriers to widespread implementation [208,209]. To address this, it was simplified into the International Clinical Diabetic Retinopathy (ICDR) severity scale, which has become the most widely adopted framework for routine clinical use globally [209,210]. In this process, the 14 levels of the ETDRS scale were condensed into 5 more accessible stages within the ICDR scale (Table 3) [210,211,212,213].

## 5. Current Therapeutic Management of Diabetic Retinopathy

The foundation for clinical management of DR is based on findings from a series of studies available on the DRCR Retina Network (DRCR.net) [216]. Management of DR relies on disease classification, in which the severity of retinopathy and the presence of DME guide clinical decisions. Depending on symptom severity and the patient’s history, DR will be managed with anti-VEGF therapy and/or PRP (Table 4). Nevertheless, the most important aspect of DR management is glycemic control.

### 5.1. Management of Diabetic Retinopathy with DME

Anti-VEGF therapy, including bevacizumab (Avastin), ranibizumab (Lucentis), aflibercept (Eylea), and faricimab (Vabysmo), is the first-line treatment for DME and neovascularization. DME can occur in any stage of DR, and anti-VEGF injections are indicated for DME only when visual acuity (VA) is 20/32 or worse [193,224,225]. In patients with good VA—defined as VA 20/25 or better—observation is indicated, and treatment should only be commenced if VA worsens [226,227]. Guidelines recommend a treat-and-extend approach, starting with monthly injections (every 4 weeks) and gradually lengthening the injection interval if DME does not worsen between injections. The choice of anti-VEGF agent should be personalized to the patient, considering the cost of care, efficacy, and patient history. In cases of refractory DME, second-line treatment consists of steroid implants or intravitreal injections. Dexamethasone implants have been shown to be superior to anti-VEGF agents in refractory cases of DME; however, their adoption as a first-line therapy is tempered by a less favourable side-effect profile, consisting of increased cataract progression and increased intraocular pressure [193,228,229,230]. In severe cases of DME with vitreomacular traction, vitrectomy is a potential second-line treatment with increased VA outcomes, while also reducing the risk of retinal detachment [231].

### 5.2. Management of Diabetic Retinopathy Without DME

In patients without DME, management relies on the severity of DR (Table 3). Intervention is only promptly required in high-risk PDR. PRP or anti-VEGF perform similarly in managing PDR, and the decision is based on clinical context [232]. If neovascularization is present, initial treatment with anti-VEGF is favoured to decrease the proinflammatory state, followed by PRP. PRP consists of performing laser burns in the retina to decrease metabolic demand, thereby reducing ischemia-driven stimulus for VEGF production while sparing the macula to preserve central vision [233]. The treatment decision should be guided by the patient’s risk of loss to follow-up. In cases where this risk is high, PRP should be performed since anti-VEGF therapy requires continuous follow-up and injections. Surgery, specifically vitrectomy, is only indicated in cases of refractory vitreous hemorrhage, tractional retinal detachment, or cataracts.

## 6. Novel Diagnostic Tools

Emerging technologies are transforming DR management by enhancing diagnostic precision and treatment personalization. Artificial intelligence (AI) and liquid biopsies are novel strategies that aim to address the current limitations in the imaging, classification and management of DR. Together, these innovations promise earlier detection, targeted therapies, and optimized resource allocation, marking a shift towards precision medicine in DR care. This section will explore both the role of liquid biopsies and AI in DR in depth.

### 6.1. Artificial Intelligence

AI revolutionizes DR management by enabling automated screening, lesion detection, and affordable prognostics. Modern AI systems excel at extrapolating retinal fundus images [234]. Fundus photography enables the detailed evaluation of microvascular alteration and structural defects that are central to the diagnosis and management of DR and several other notable retinal diseases [235]. Fundus photography remains the foundation of AI-assisted DR screening, and convolutional neural networks (CNNs), which are deep learning models designed to read medical images, enable automated lesion detection, grading severity, and microaneurysm, hemorrhage, and exudate detection at human-expert-level performance (>90% sensitivity/specificity), as demonstrated in numerous groundbreaking publications [236]. IDx-DR (FDA-approved in 2018) employs CNNs developed and validated to analyze retinal images, identify characteristic DR lesions, and subsequently categorize the disease. It can diagnose more-than-mild DR (mtmDR) with 87.2% sensitivity and 90.7% specificity [237]. Its successor, IDx-DR X2.1, employs multiple CNNs that can classify fundoscopy results into four categories, incorporating prognosis (negative, referable DR, vision-threatening DR, or low image quality) while also maintaining good performance across ethnic and image quality variations [238]. EyeArt v2.2.0 (FDA-approved in 2020), a cloud-based retinal diagnostic software device trained to analyze fundus images, identifies mtmDR (95.5% sensitivity, 85% specificity) and vision-threatening DR (92% sensitivity, 94% specificity) simultaneously [239]. EU-approved models like SELENA+ (developed using multi-ethnic datasets for glaucoma and age-related macular degeneration detection) and RetmarkerDR (microaneurysm turnover monitoring), trained to diagnose DR based on fundus images at different time points in a patient’s disease progression, enhance screening performance in diverse populations [240,241]. Novel systems, such as DeepDR Plus, use 5-year progression risk and extend screening intervals to 32 months [242].

As the most extensively studied modality, fundus photography-based AI models continue to evolve, addressing the pressing need for scalable, guideline-recommended yearly screenings [243]. Challenges still exist, including (i) data quality with images primarily impacted by low resolution and cataracts, which necessitates human intervention, (ii) algorithmic bias that requires more advancements to enhance generalizability across diverse ethnic populations, and (iii) infrastructure expense, such as cloud servers and high-resolution cameras, which hinders uptake in resource-limited areas [240].

Beyond fundus photography, AI is also being applied to OCT and FA to improve diagnostic and management accuracy for DR subtypes such as DME. OCT-based CNNs (e.g., VGG16, ResNet-50) can segment retinal layers accurately and quantify 3D biomarkers, such as macular thickness and intraretinal fluid, to non-invasively diagnose DME subtypes, including diffuse retinal thickening (DRT), cystoid macular edema (CME), and serous retinal detachment (SRD), that are known to be predictive of anti-VEGF therapy response (e.g., SRD requires more injections than DRT). Although OCT remains the gold standard for DME, its cost limits scalability; thus, many deep learning models have paired OCT-confirmed DME diagnoses with fundus photographs to improve diagnostic accuracy [244,245]. Furthermore, FA-driven AI can quantify vascular morphology and microaneurysms [246,247]. Finally, smartphone-based AI, such as Medios AI [248] and Remidio Fundus on Phone, [249] has been deployed and has achieved reliable accuracy for referable DR at reduced costs.

The rise of big data in medicine, driven by comprehensive electronic health records (EHRs), has empowered AI-driven multimodal models to transform disease management, including DR. These models unify retinal imaging, proteomics, metabolomics, genetic data, and EHRs to advance precision medicine in DR care. While AI applications in DR beyond imaging-based screening and early detection remain limited, emerging studies demonstrate promising advances in predicting anti-VEGF therapeutic response [250], personalizing injection regimens [251,252], forecasting progression via glucose dynamics [253], identifying comorbidities through EHR-based and imaging risk stratification [254,255], prioritizing screening urgency via clinical biomarkers [256], and decoding molecular mechanisms via liquid biopsy proteomics [257]. This integration of big data will usher in a transformative era of precision-driven, data-informed ophthalmic care.

### 6.2. Liquid Biopsies and miRNA

**Figure 6:**
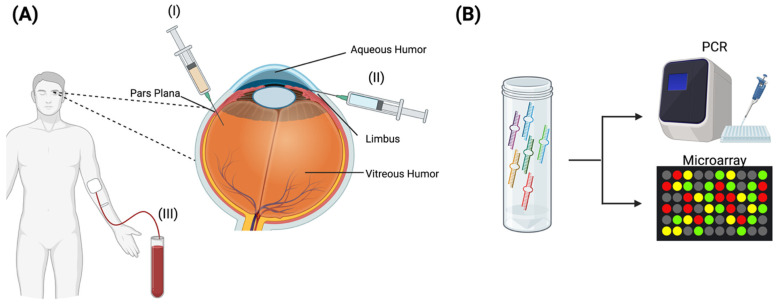
Illustration of sampling sites for liquid biopsy analysis in the management of diabetic retinopathy. (**A**) Sampling of fluids for liquid biopsy in DR is primarily performed using three methods. (I) Vitreous Humour sampling through the pars plana, (II) Aqueous Humour sampling through the limbus paracentesis and (III) Blood collection. (**B**) miRNA is purified from the samples and examined with PCR or microarray analyses. Created in BioRender. Tuli, N. (2025) https://BioRender.com/nqoo9ze.

Liquid biopsy is a breakthrough innovation in DR management that involves testing biomarkers in body fluids such as blood, aqueous humour (AH), and vitreous humour (VH) [257,258,259,260]. It is especially valuable for real-time monitoring of disease activity for response to therapy without the risks of conventional tissue biopsies [261]. In DR, the main ocular fluids sampled are AH and VH, which are enriched with disease-specific biomarkers (Figure 6) [258,262]. AH sampling via paracentesis is a viable indication of retinal pathology, since intraocular fluids from the vitreous–carrying specific molecules, such as VEGF, cytokines, and other biomarkers - diffuse to the anterior chamber [258,263]. AH sampling can serve as a viable surrogate for assessing retinal pathology. It is considerably safer and less invasive than VH collection, which is performed only during surgery for specific clinical indications [258]. Nevertheless, both fluids shared similar biomarker profiles (e.g., VEGF, IL-6) associated with DR severity, and can be sampled for specific biomarkers [262].

In DR, liquid biopsy can detect key markers, including cytokines, growth factors, metabolites, and microRNAs (miRNAs). While specific metabolites, such as glutamate, and proteins, including cytokines and angiogenic factors, have been identified and correlated with DR severity, the most promising molecule in AH biopsies is miRNA [257,264,265]. miRNAs are small (~22 nucleotides), non-coding RNAs that regulate gene expression post-transcriptionally by binding complementary sequences in the 3’ untranslated region (3’ UTR) of target mRNAs, leading to their degradation or translational repression [266]. They are synthesized by sequential processing through Drosha and Dicer enzymes, with the end products modulating cellular processes, including proliferation, angiogenesis, inflammation, and apoptosis, all of which are crucial in DR pathogenesis [259,266,267]. Among the identified biomarkers, miRNAs stand out for their stability, specificity, and sensitivity in reflecting the disease’s molecular profile. Encapsulated in exosomes or protein complexes, miRNAs are resistant to RNase degradation, making them ideal for long-term monitoring and potential future therapeutic delivery modality [268,269]. A meta-analysis found that miRNAs exhibited excellent diagnostic capability for DR (sensitivity: 0.82, specificity: 0.84, AUC: 0.90), and panels of multiple miRNAs significantly enhanced sensitivity, indicating their potential as effective biomarkers [269]. Notably, this study assessed miRNAs in AH and serum samples, with serum samples exhibiting higher detection accuracy [269]. Overall, there is strong evidence that miRNAs from both serum and AH are promising biomarkers for DR.

Over 50 miRNAs have been found to be associated with DR, with each adding to a better understanding of the pathophysiology of DR subtypes [269,270,271,272]. Studies have shown that circulating miRNAs, particularly miR-210, can be used to assess the severity and stage of DR, with higher levels associated with a greater risk of PDR [273]. Alternatively, certain miRNAs, including miR-27b and miR-320a, are specific to DR in type 1 diabetic patients, whereas others are specific to DR in type 2 diabetics, demonstrating distinct miRNA profiles reflecting different DR pathologies [274,275]. In addition, the miRNA profile can reflect the underlying pathophysiology, with some, such as miR-126, which suppresses the inhibitors of the VEGF pathway, indicative of angiogenic dysfunction [270,276]. While others, such as miR-320a and miR-200b, reflect pathways integral to DR pathology, including inflammatory and cell proliferation pathways [270]. Thus, specific miRNA profiles can provide insight into the molecular abnormalities of the retina in DR. Finally, results from liquid biopsy can also predict anti-VEGF response. Notably, VH-specific miR-23b-3p is downregulated in anti-VEGF PDR responders, suggesting its absence may be a predictor of response to anti-VEGFs [277]. Additionally, lower post-injection serum TGF-β1 levels were indicative of a response to anti-VEGF treatment in patients with NPDR, suggesting that cytokines can also serve as biomarkers to predict response to therapy [278]. Ultimately, liquid biopsy assessing for DR biomarkers offers three promising benefits to DR management. The first is that they may enable early disease monitoring, identifying abnormal miRNAs specific to pre-clinical DR while also providing biomarkers of more severe disease. Secondly, they enable monitoring and prediction of disease progression, as specific miRNAs associate with specific pathways and more severe disease forms. Lastly, precise miRNA profiles may predict responses to specific anti-VEGFs, enabling personalized treatment to improve patient outcomes.

Despite the promise of liquid biopsy as a diagnostic tool for DR, several gaps remain to be addressed before its real-world clinical application. In general, the novel diagnostic method requires validation across diverse populations and disease severity (DME, NPDR, and PDR). Additionally, standardization of procedures for sample acquisition and RNA processing will be vital to ensure reliability. Over time, if these gaps are addressed, liquid biopsy could become an additional diagnostic tool for stratifying and monitoring DR.

## 7. Recent Advances in Diabetic Retinopathy Treatment

PRP and anti-VEGF therapy have proven to be effective treatments for DR, preventing severe vision loss. However, they do have limitations, as they are invasive and, in the case of anti-VEGF therapy, require frequent intravitreal (IVT) injections. Currently, new treatments under study target the VEGF pathway, but with the goal of improving care by reducing treatment burden for patients while also improving visual outcomes.

Additionally, several studies are investigating agents targeting other novel pathways integral to DR pathogenesis. This section will summarize and discuss the current advances in DR treatments.

### 7.1. Novel VEGF-Targeting Agents

IVT injectable therapies, predominantly anti-VEGFs, are the gold standard for the treatment of CI-DME and can also be used for PDR. IVT injections have been transformative as they enable localized administration of anti-angiogenic agents to the retina, thereby preventing systemic adverse effects. As they remain at the forefront of clinical management of DR, several ongoing clinical trials are examining new anti-VEGFs with the goal of lengthening treatment intervals or improving clinical outcomes (Table 5). Some of the most promising novel anti-VEGF agents will be discussed in the paragraphs below.

KSI-301 (Kodiak Sciences), named tarcocimab tedromer, is an antibody polymer conjugate that targets VEGF to treat DME primarily. Its use in patients with DME was suboptimal, performing inferiorly in comparison to aflibercept at improving BCVA [290]. Despite its limitations in DME, KSI-301 has shown promise in treating NPDR, as 41.1% of patients receiving KSI-301 achieved a ≥2-step improvement in the Diabetic Retinopathy Severity Score (DRSS) compared to 1.4% of shams [291]. New studies are underway to extend the interval between KSI-301 injections to 6 months, potentially providing a promising treatment for patients with NPDR without DME [292].

RC28-E (RemeGen), a dual decoy receptor targeting both VEGF and basic fibroblast growth factor (bFGF), is also under study [293]. Preclinical studies have demonstrated that RC28-E has protective effects in early DR [294]. This led to a Phase 2 study investigating RC28-E in patients with moderate-to-severe NPDR (NCT04782128) and a Phase 3 study comparing RC-28E with aflibercept in patients with DME (NCT05885503).

Another anti-VEGF being studied is OPT-302 (Opthea Limited), a recombinant fusion “trap” molecule that consists of three ligand-binding sites specific to VEGF-C and D, sequestering them and preventing their binding to VEGFR-2 and 3. Since it does not target VEGF-A, the primary VEGF molecule involved in DR, it is intended for use in combination with aflibercept [295]. A Phase 1 study (NCT03397264) examining the combination therapy in patients with persistent CI-DME that has suboptimal response to anti-VEGF-therapy found that mean change in BCVA in from the combination therapy was +7.7 letters (95% confidence interval: 2–13.3) from baseline, showing this combination therapy may be a possible therapy for patients with suboptimal response to current anti-VEGFs [280].

Integrins have emerged as targets for novel therapies, as they are vital co-receptors for VEGF receptors [296]. Several agents targeting integrins, including AG-73305 (Allgenesis Biotherapeutics), a bispecific fusion Fc protein targeting both VEGF and integrin pathways. Interim Phase 2 results (NCT05301751) showed AG-73305 improved BCVA and CST in DME patients previously treated with anti-VEGF therapy [281]. Another integrin-targeting agent is OCU200 (Ocugen), a bispecific recombinant fusion protein composed of tumstatin that inhibits integrin coreceptors and transferrin, localizing the agent to transferrin receptors expressed on retinal and choroid endothelial cells [297]. OCU200 is being evaluated in a Phase 1 trial (NCT05802329) as monotherapy and in combination with ranibizumab for CI-DME.

### 7.2. Therapies Targeting Novel Pathways

**Figure 7:**
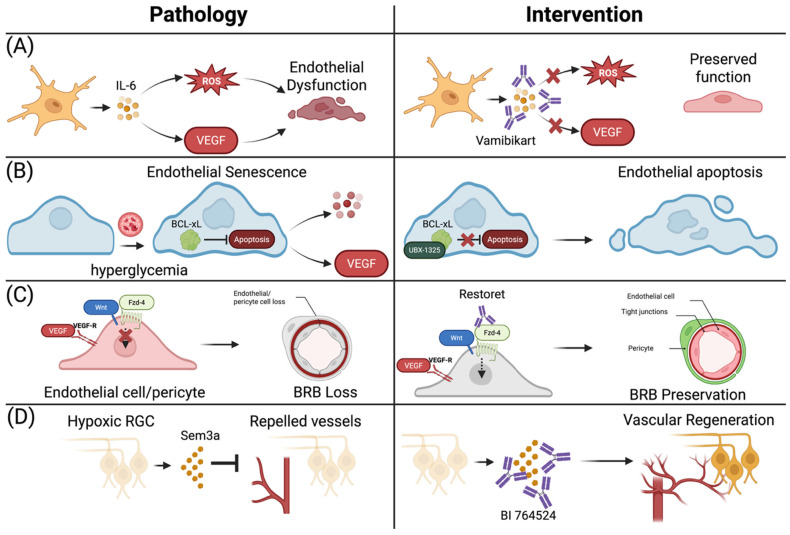
Novel therapeutic pathways for the management of diabetic retinopathy. New therapies targeting non-VEGF pathways are under investigation. The underlying pathology and mechanism of intervention are illustrated. (**A**) Preserving endothelial function with vamikibart. IL-6, released by immune cells, drives ROS and VEGF production, which leads to endothelial dysfunction. Vamikibart is an IL-6-specific antibody that inhibits its activity, preserving endothelial function. (**B**) Endothelial senescence is reversible with UBX-1325. Senescence of endothelial cells allows them to survive and produce cytokines and VEGF. UBX-1325 inhibits BCL-xL, thereby preventing apoptosis and allowing activation of apoptosis and elimination of endothelial cells. (**C**) Restoret^TM^ drives Wnt-signalling to preserve the BRB. VEGF signalling causes BRB compromise. By antagonizing the pathway, Restoret^TM^ (EyeBio/Merck, London, UK) preserves BRB integrity. (**D**) Reversing retinal ischemia with BI 764524. Hypoxic RGCs release Sem3a, inhibiting normal vascular growth. BI 764524 inhibits Sem3a, enabling proper vascular regeneration to address retinal hypoxia. Created in BioRender. Tuli, N. (2025) https://BioRender.com/5nk6uck.

New therapies for DR are now targeting novel pathways to palliate for the limitations of targeting VEGFs alone. As our understanding of DR pathology has grown, many new viable targets have emerged. These include new agents targeting inflammatory pathways, senescence, ischemia, and wingless-related integration site (Wnt) pathway, all of which are currently being investigated in clinical studies (Figure 7).

Cytokines, including interleukins (ILs), are soluble proteins released by immune cells that mediate inflammatory and immune responses. IL-6 is a cytokine central to inflammatory responses and has been shown to be elevated in DR, triggering vascular endothelial cell dysfunction through oxidative stress pathways and VEGF production, leading to DME [298]. IL-6 inhibitors have also shown promise in treating treatment-resistant macular edema [299]. Vamikibart (RG6179, Roche) is a monoclonal antibody that directly inhibits IL-6 activity. A Phase 1 study (NCT06771271) demonstrated that IL-6 inhibition with vamikibart was safe and tolerable in patients with DME, leading to two new studies examining vamikibart as a monotherapy and in combination with ranibizumab (NCT05151731/NCT05151744) [300].

In diabetic patients, hyperglycemia induces endothelial cells to enter a senescent state, a period during which cells do not divide but remain functional [301]. Senescent cells remain viable in DR, continuing to produce inflammatory factors and VEGF that contribute to the breakdown of the BRB and promote angiogenesis, respectively [285,302]. UBX1325, named foselutoclax (Unity Biotechnology, Inc.), is a novel small molecule targeting senescent cells through B Cell lymphoma-xL (BCL-xL) inhibition. BCL-xL is a member of the antiapoptotic BCL-2 family whose function is anti-apoptotic [303]. Preclinical studies have shown that small-molecule inhibition of BCL-xL induces senolysis, thereby reducing angiogenesis [302]. In a Phase 1 trial, a single IVT injection of UBX1325 was safe and tolerable and yielded a mean improvement of 9.5 ± 4.4 ETDRS letters at 24 weeks, showing promise as a therapy for DR [285].

The Wnt pathway is also being investigated as a therapeutic target, as it has been shown to be critical for retinal vascular development [304]. MK-3000 (Restoret^TM^, previously EYE-103, EyeBio/Merck, London, UK) is a tetravalent, tri-specific antibody administered by intravitreal injection that targets Wnt, with the goal of preserving the BRB and preventing vascular leakage [297]. It mimics the natural Wnt ligand Norrin, which agonizes Wnt signalling through Frizzled 4 (Fzd-4) to maintain tight junction organization in the BRB, maintaining vascular integrity [305,306,307]. Mouse models deficient in Norrin exhibit a pathology very similar to DR, and administration of Norrin can potentially restore the BRB [304,308]. Currently, two clinical trials, AMARONE (NCT05919693) and BRUNELLO (NCT06571045), are underway to investigate Restoret^TM^ in patients with DME.

The final noteworthy novel pathway being therapeutically targeted is Semaphorin 3A (Sema3A). Sema3A is a guidance signal secreted by hypoxic retinal ganglion cells in response to inflammatory cytokines. It repels new blood vessels away from ischemic retinal tissue, causing vascular leakage [309,310]. Preclinical studies have shown that silencing Sema3A can enhance vascular regeneration, preventing the disorganized neovascularization observed in DR [309,310]. BI 764524 (Boehringer Ingelheim) is an anti-Sema3A antibody that reduces ischemic areas in the retina of mice with oxygen-induced retinopathy [311]. Currently, BI 764524 is being studied in patients with diabetic macular ischemia (DMI) [312]. Given the lack of established therapies for DMI, this study offers a potentially important advancement for this patient population.

### 7.3. Implants and Inserts

Anti-VEGF therapy is highly burdensome due to the requirement of frequent IVT injections. As a result, new strategies are being examined that would reduce the burden of monthly or bimonthly injections. Currently, several studies are investigating implants that can provide sustained delivery of anti-VEGF and other molecular therapies to extend drug exposure, thereby reducing the need for frequent injections (Table 6).

**Figure 8:**
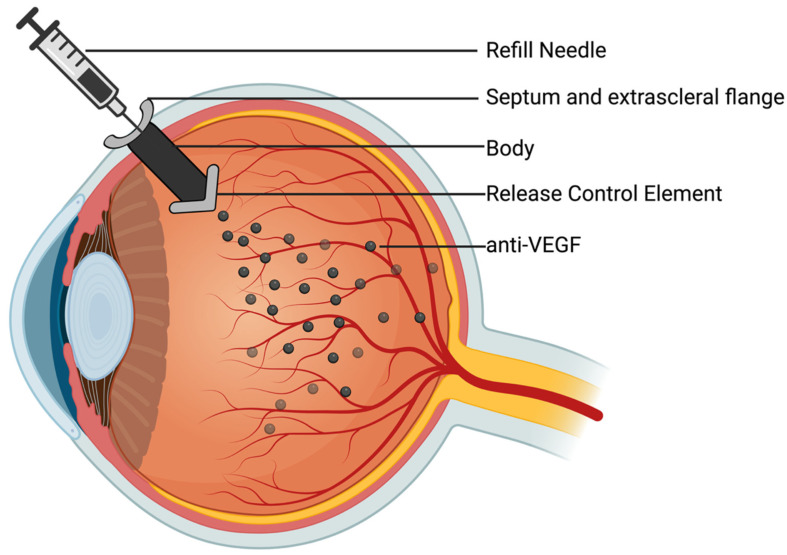
Schematic of portal delivery system implant. A surgical procedure makes an incision through the pars plana. The implant is inserted with the release-control element facing the retina to enable sustained, long-term drug release. The septum and extrascleral flange anchor the implant to the sclera and conjunctiva, providing an access point for in-office refill. Created in BioRender. Tuli, N. (2025) https://BioRender.com/u7ff907.

The portal delivery system (PDS) with ranibizumab, named Susvimo^®^ (Genentech/Roche, San Francisco, CA, USA), is the first anti-VEGF delivery implant approved by the FDA for AMD back in 2021 (Figure 8) [313]. The PDS is a non-biodegradable, refillable implant that can deliver a several-month supply of ranibizumab directly into the vitreous. Insertion of the implant requires surgery under local anesthesia, during which a small incision is made in the sclera and pars plana to position the implant so that its open mouth faces the vitreous and the external side resides outside the sclera [313,314]. In February 2025, Susvimo^®^ received FDA approval for DME following the positive results of the Phase 3 PAGODA study (NCT04108156, Table 7). The study found that best-corrected visual acuity (BCVA) from baseline over 60-64 weeks with a Susvimo^®^ implant requiring refill exchange every 24 weeks (increase of 9.6 letters) was non-inferior to monthly ranibizumab (increase of 9.4 letters) [315]. Adverse events, including vitreous hemorrhage and implant dislocation, were more common in the PDS arm (27.5%) than the ranibizumab group (8.9%). However, there were no reported cases of severe complications, including endophthalmitis or retinal detachment [315]. The approval of Susvismo^®^ is transformative for patients with DME, offering an alternative treatment to monthly injections that significantly reduces treatment burden.

Suvismo was investigated in patients with NPDR, without DME (NCT04503551). 80.1% of patients who received the PDS showed a 2-step improvement in DRSS from baseline, compared with 9.0% of controls who received no intervention. The PDS arm also had a lower rate of development of DME, PDR, or anterior segment neovascularization (ASNV) (7.1%) through 52 weeks compared to controls (47.0%). However, there were increased adverse events in the Suvismo arm, including cataract (6.7%), vitreous hemorrhage (5.7%) and retinal detachment (1%) [316]. Thus, Suvismo offers a new therapy for patients with NPDR without DME. Nonetheless, these benefits must be weighed against the risks of adverse events associated with the implantation surgery.

OTX-TKI, named Axpaxli^TM^ (Ocular Therapeutix Inc., Bedford, MA, USA), is an intravitreal, bioerodible, hydrogel implant under study. It delivers axitinib, a highly selective inhibitor of all VEGF and PDGF receptors for 6 months or longer. Initially developed to inhibit tumour angiogenesis, axitinib has been repurposed to treat DR, with preclinical studies showing it can inhibit the VEGF receptor pathway and reduce oxidative stress in retinal endothelial cells exposed to high glucose [317]. Currently, OTX-TKI is being studied in patients with moderate-to-severe NPDR in the Phase 1 HELIOS study (NCT05695417). One-year results from the study showed that 23.1% of treated patients had a 2-step or greater improvement in DRSS at 48 weeks, compared with 0% in the sham control arm. No patients progressed to PDR or CI-DME through 48 weeks compared with the sham control arm, in which 37.5% of patients developed PDR or CI-DME through 48 weeks, demonstrating that OTX-TKI may reduce the rate of DR progression [318].

Another implant, EYP-1901 (Duravyu^TM^, EyePoint Pharmaceuticals, Watertown, MA, USA), is a bioerodible intravitreal insert that enables the sustained release of vorolanib, a selective tyrosine kinase and pan-VEGF receptor inhibitor [319,320]. It uses Durasert^®^ technology (EyePoint Pharmaceuticals, Watertown, MA, USA), an FDA-approved method administered via IVT injections that allows the sustained release of vorolanib for approximately 9 months with a single IVT injection [321]. EYP-1901 has been studied as a treatment for DR in two trials: the Phase 2 VERONA study (NCT06099184) in patients with DME and the Phase 2 PAVIA study (NCT05383209) comparing EYP-1901 to aflibercept in patients with NPDR. The PAVIA trial found that EYP-1901 reduced rates of NPDR progression; however, it did not meet its primary endpoint of improving DRSS [322]. The VERONA trial’s interim results demonstrated a +8.9-letter improvement in BCVA for the EYP-1901 arm, compared with a +3.2-letter improvement in the aflibercept arm, suggesting EYP-1901 may be a promising therapeutic for patients with DME [323].

The PER-001 IVT implant (Perfuse Therapeutics, San Francisco, CA, USA) is a bioerodible device delivered to the vitreous cavity using a 25-gauge applicator. Once implanted, it provides sustained release of PER-001, a small-molecule endothelin receptor antagonist. PER-001 implants have been demonstrated to be safely administered and have shown efficacy in animal models of oxygen-induced retinopathy [324,325]. Currently, PER-001 is being studied in patients with moderate-severe NPDR (NCT06003751).

A final noteworthy implant is AR13503, is a bioerodible implant which releases Rho-kinase (ROCK) and PKC inhibitors over 4–6 months. Both the ROCK and PKC pathways are upregulated in diabetes, which can promote angiogenesis, and their inhibition has been validated as a promising therapeutic strategy in preclinical models [326,327]. Currently, AR-13503 is being studied in patients with AMD and DME (NCT03835884).

### 7.4. Gene Therapies

Gene therapy can also address the limitations of IVT anti-VEGF injections by enabling retinal cells to express anti-VEGFs endogenously. This is achieved by injecting a viral vector, most commonly an adeno-associated virus (AAV), that contains a transgene encoding an anti-VEGF. The AAV delivers the transgene to retinal cells, which will then express an anti-VEGF continuously, with the goal of achieving long-lasting effects that eliminate the need for monthly IVT injections. Gene therapy has shown immense promise in preclinical studies. However, there has yet to be a gene therapy that has shown definite promise in clinical settings. Currently, several clinical studies are investigating novel gene therapies in patients with various forms of DR (Table 8).

4D-150 (4D Molecular Therapeutics) is a R100 vector that delivers a transgene encoding both aflibercept and a VEGF-C inhibitory RNA interference (RNAi). It employs a vector, a modified AAV, that has been shown to achieve superior transduction of human retinal cells compared to wild-type AAV [328]. It encodes aflibercept and an RNAi, which together inhibit VEGF A, B, C and placental growth factor (PIGF) [329]. A single IVT injection of 4D-150 was shown to be safe in patients with exudative AMD, and it is currently under study in patients with DME (NCT05930561) [330,331].

RGX-314 (REGENXBIO, AbbVie) is an AVV8 vector containing a transgene encoding ranibizumab that needs to be administered in the subretinal space through vitrectomy surgery. RGX-314 was first studied in neovascular AMD in several clinical trials, which found that subretinal and suprachoroidal delivery of RGX-314 was tolerated and led to sustained gene expression, resulting in improved BCVA and central retinal thickness [332,333]. These promising results have led to its study in patients with NPDR or PDR without CI-DME (NCT04567550). Interim results have shown that 100% of eyes demonstrated stable disease improvement, with 70.8% of patients receiving RGX-314 achieving a > 1-step improvement in the DRSS, compared to 25.0% in the observational control arm [334].

Lastly, ADVM-022 (Adverum Biotechnologies, Inc., Redwood City, CA, USA) is an AAV vector modified to express aflibercept and optimized for intravitreal injection. In preclinical studies, a single dose of ADVM-022 was shown to be effective in treating laser-induced choroidal neovascularization (a model of wet age-related macular degeneration) in non-human primates and to result in sustained aflibercept expression 30 months after injection. ADVM-022 was studied in the Phase INFINITY study (NCT04418427); however, it was halted prematurely due to adverse events, including rapid hypotony and other clinically significant decreases in intraocular pressure [335].

While gene therapies have strong theoretical promise, their integration into clinical practice has been slow to unfold. There are several reasons for the failure of gene therapies, including patient selection, our limited understanding of pathophysiology, adverse reactions, the therapy’s own toxicity, and the limited packaging capacity of viral vectors [336]. Gene therapies have shown more promise for monogenic disorders; however, as our understanding of gene therapy and its delivery grows, so may their clinical success.

## 8. Conclusions and Challenges

Significant limitations persist in the diagnosis, therapeutic management, and prognostication of DR. The existing anti-VEGF therapies, while helpful for many patients, require repeated administration, pose risks of off-target effects on non-endothelial cells, and fail to address the multifactorial pathogenesis of DR, including VEGF-independent pathways [337]. A considerable percentage of DR patients are non-responders or have suboptimal responses to anti-VEGF therapy, demonstrating the urgent need for personalized therapeutic strategies [338]. The “one-size-fits-all” concept of DR treatment inadequately accounts for disease heterogeneity among patients [258,339]. This limitation mirrors the overall challenges in diabetes care, in which precision medicine has emerged as a transformative paradigm [339].

Looking ahead, the future of DR management will likely centre around personalized medicine. Integrating profiling with advanced imaging and artificial intelligence will enable earlier diagnosis, refined risk stratification, and individualized therapy. Precision diagnostics, such as liquid biopsy and AI-assisted imaging, may soon enable real-time monitoring of disease activity and tailored treatment regimens. Meanwhile, gene therapy and sustained drug delivery systems are poised to reduce treatment burden and improve patient adherence. As our understanding of DR pathogenesis deepens, therapeutic strategies will expand beyond VEGF inhibition to target upstream events in disease development. Ultimately, bridging these translation gaps will be essential to advance DR care.

## Figures and Tables

**Table 2:** Overview of the diagnostic tools available for the diagnosis of diabetic retinopathy.

Diagnostic Tool	Purpose	Expected Findings	Strengths and Limitations	References
Hemoglobin A1C	Systemic risk assessment for DR onset and progression. Indicates glycemic control.	Elevated HbA1c (greater than 7%) correlates with increased DR severity	Strengths: Guides systemic treatment Non invasive Limitations: Does not reflect retinal pathology Only assess risk factors	[188,189]
Fundus examination	Primary screening and diagnostic tool for DR Allows for grading of severity and presence of DME	NPDR: microaneurysm hemorrhage hard exudates cotton-wool spots PDR: neovascularization of the disc (NVD) or elsewhere (NVE) DME: Central retinal thickening Lipid exudates at the macula	Strengths: Widely accessible, low cost Visualize the entire retina Limitations: Subjective/examiner dependent. Limited ability to quantify edema or ischemia. Peripheral lesions can be missed.	
Fluorescein angiography	Angiographic tool to assess retinal perfusion, microvascular leakage and guide laser treatment	Hyperfluorescent spots: Diabetic macular ischemia (DMI) Capillary nonperfusion (dropout zones). Microaneurysms leaky vessels highlighted by fluorescein leakage. NVD/NVE with fluorescein leakage in late frames	Strengths: Sensitive for ischemia and leaky neo vessels Gold-standard for detecting retinal capillary non-perfusion Limitations: Invasive: intravenous dye can cause nausea and allergic reactions Requires multiples images over several minutes. Limited visualization of deep retinal and choroidal vessels	[190,191,192,193]
Optical coherence tomography	High-resolution cross-sectional imaging of retinal layers Essential for diagnosis and monitoring DME	Retinal thickening in the macula Cystoid spaces in intraretinal layers Subretinal fluid or pigmented epithelium detachment Detects microstructural changes associated DMI Hyperreflective foci: hard exudates	Strengths: Non-invasive, rapid and quantitative Provides objective retinal thickness measurement and imaging biomarkers Limitations: Relatively limited field of view Sensitive to motion artefacts and segmentation error Does not catch vascular leakage	[194,195]
Optical coherence tomography—angiography	A non-invasive angiographic imaging of retinal microvascular using OCT signal decorrelation Used in clinical practice to assess capillary dropout and neovascular networks without dye	Enlarged foveal avascular zone (FAZ) and capillary density loss Microaneurysms as focal flow disturbances DMI visualized as decreased capillary perfusion	Strengths: Non-invasive, rapid and quantitative Three-dimensional, providing depth of visualization Detects early microvascular changes Limitations: Relative limited field of view Sensitive to motion artifacts and segmentation error Does not catch vascular leakage	[194,196]
Indocyanine green angiography	Imaging of choroidal circulation and deeper retinal vessels Adjunctive imaging used in complicated cases	Hypofluorescent spots: areas of non-perfusion Hyperfluorescent spots: choroidal leakage or microaneurysms	Strengths: Detects choroidal abnormalities Limitations: Invasive Adverse allergic reactions to dye Contraindicated in pregnancy and liver disease	[197,198,199]
B-mode ultrasound	Sonographic imaging used when media is opaque, to assess for DR complications	Detects: Dense vitreous hemorrhage Cataracts Tractional Retinal Detachment	Strengths: Quick and non-invasive Detects severe complications Limitations: Limited detail of retinal vasculature or fundus appearance Operator-dependant	

**Table 3:** Classification of diabetic retinopathy and indications for interventions.

ETDRS	ICDR Severity Score	Findings on Fundoscopy	Indicated Intervention	References
Diabetic Retinopathy				
No retinopathy	No retinopathy	Diabetic without retinal manifestations	Observation	
Very Mild NPDR	Mild NPDR	Microaneurysm only	Observation with 12-month follow-up	
Mild, Moderate, Moderately Severe NPDR	Moderate NPDR	Any of: Microaneurysms Retinal dot or blot hemorrhage Hard exudates or cotton wool spots No signs of severe NPDR	Observation with 12-month follow-up	[4,193,210]
Severe NPDR	Severe NPDR	The “4-2-1 Rule” ≥20 intraretinal hemorrhages in all 4 quadrants Definite venous beading in ≥2 quadrants Prominent intraretinal microvascular abnormalities in ≥1 quadrant	PRP reduces risk of severe vision loss, OR: Deferral of treatment until development of high risk characteristics	
Mild, Moderate, High-risk, or Advanced PDR	PDR	At least one of: Neovascularization of disc (NVD) Neovascularization elsewhere (NVE) Vitreous or pre-retinal hemorrhage	PRP Anti-VEGF Both perform similarly in reducing risk of severe vision loss	[193,210,212]
DME severity scale				
No DME		No retinal thickening or hard exudates	Observation	
Noncentre-involved DME (NCI-DME) Centre-involved DME (CI-DME)	Mild DME	Some retinal thickening and hard exudates distant from centre of the macula	Observation, if progressing: Focal Macular laser	
	Moderate DME	Retinal thickening and hard exudates approaching centre of the macula but not involving it		[210,214,215]
	Severe DME	Retinal thickening or hard exudates involving the centre of the macula	3. Anti-VEGF 4. Steroid implant 5. Focal macular laser	[210]

**Table 4:** Current treatment modalities for diabetic retinopathy.

Therapeutic Modality	Mechanism of Action	Indications	Frequency	Side Effects	References
Intravitreal anti-VEGF injections	Antibodies or fusion trap proteins that bind VEGF-A preventing it from interacting with its receptor	Indicated in DR with centre-involving CI-DME Considered in non-centre-involving (NCI)-DME Considered in high-risk NPDR without CI-DME Alternative or adjunct to PRP in PDR without CI-DME	Treat-and-extend method with injections every 4–12 weeks at regular intervals	Endophthalmitis: ~1% Uveitis and ocular inflammation: 1–2% Floaters Acute elevated IOP Ocular hemorrhage Retinal vessel changes Cataracts Retinal Detachment: 0–0.6%	[217,218]
Panretinal Photocoagulation	Either an argon green or blue-green laser is used to apply at least 1200 laser spots of 500μ microns in diameter to reduce tissue ischemic drive	Indicated in macula-sparing DR Indicated in high-risk PDR Considered in severe NPDR and non-high-risk PDR	Single session or spread out in multiple sessions if patient is sensitive to the procedure.	Patient discomfort Peripheral visual field constriction Worsened night vision Worsened DME Temporary central vision loss Vitreous hemorrhage Pupil dilation (mydriasis)	[195,219,220,221]
Focal laser photocoagulation	Laser therapy targeting microaneurysms directly, at least 500 microns away from the fovea	Considered in NPDR with NCI-DME	Single session or spread out in multiple sessions if patient is sensitive to the procedure.	Central scotomas in cases where laser burns are too close to fovea “Laser creep”: expansion of the laser scar Choroidal neovascularization and subretinal fibrosis	[195,222,223]
Intravitreal corticosteroids	Intravitreal injection or implant of steroids, either dexamethasone, triamcinolone acetonide or fluocinolone acetonide	2. Indicated as second-line treatment in cases of refractory DME 3. May be considered as first line if pseudophakic	Every 4–6 months for injectables. Every 6–36 months for longer-acting implants.	Cataract formation Cataract progression Elevated IOP in 50% of injections Endophthalmitis Hypotony from wound leak Anterior-chamber migration causing corneal endothelial decompensation	[193,224,225]

**Table 5:** Novel anti-VEGF targeting and alternative pathway targeting agents under clinical study for DR.

Clinical Trial Identification (Start Year–End Year)	Phase	Drug (Company)	Mechanism of Action	DR Stage	Study Title/Conclusion
VEGF Pathway Targeting					
NCT03790852 (2018–2022)	1	KSI-301 (Tarcocimab tedromer, Kodiak Sciences, Palo Alto, CA, USA)	Antibody-biopolymer conjugate targeting VEGF	Treatment naïve DME	**Exploratory Study to Investigate the Bioactivity, Ocular and Systemic Safety, Tolerability, and Pharmacokinetics Following Single and Multiple Intravitreal Administrations of KSI-301 in Subjects With wAMD, DME and RVO**
NCT04611152 (2020–2023)	3			Treatment naïve DME	**A Trial to Evaluate the Efficacy, Durability, and Safety of KSI-301 Compared to Aflibercept in Participants With Diabetic Macular Edema (DME) (GLEAM)**
NCT04603937 (2020–2023)	3			Treatment naïve DME	**A Study to Evaluate the Efficacy, Durability, and Safety of KSI-301 Compared to Aflibercept in Participants With Diabetic Macular Edema (DME) (GLIMMER)**
NCT05066230 (2021–2023)	3			Moderately severe- severe NPDR	**A Study to Evaluate the Efficacy and Safety of Intravitreal KSI-301 in Non-proliferative Diabetic Retinopathy (NPDR) (GLOW)**
NCT06270836 (2024–2026)	3			Any DR	**A Study to Evaluate the Efficacy and Safety of Tarcocimab Tedromer Compared With Sham Treatment in Participants With Diabetic Retinopathy (DR) (GLOW2)**
NCT04782128 (2021–2024)	2	RC28-E (RemeGen, Yantai, China)	Dual decoy receptor targeting both VEGF and basic Fibroblast Growth Factor (bFGF)	Severe NPDR	**A Randomized, Open-label, Multicenter Study of the Efficacy and Safety of RC28-E Injection in Subjects With Moderately Severe to Severe Nonproliferative Diabetic Retinopathy**
NCT04782115 (2021–2023)	2			DME	**Evaluation of RC28-E Injection in Diabetic Macular Edema** RC28-E injections yielded more significant improvements in BCVA in comparison to conbercept [279].
NCT05885503 (2023–2026)	3			DME	**A Phase III, Multicenter, Randomized, Double-blind, Active Controlled Trial of RC28-E Intravitreal Injection in Subjects With Diabetic Macular Edema**
NCT03397264 (2018–2020)	1/2	OPT-302 (Opthea, South Yarra, Australia)	Recombinant fusion protein targeting VEGF-C and D	Persistent CI-DME	**Dose Ranging Study of OPT-302 With Aflibercept for Persistent Diabetic Macular Edema** OPT-302-aflibercept combination therapy improved BCVA by 7.7 letters [280].
NCT05301751 (2022–2024)	2	AG-73305 (Allgenesis Biotherapeutics, Taipei, Taiwan)	Bi-specific Fc fusion protein targeting VEGF and integrin pathways	CI-DME	**AG-73305 Single Ascending Dose Cohort Study in DME** AG-73305 improved BCVA and CST in DME patients previously treated with anti-VEGF therapy [281].
NCT05802329 (2023-present)	1	OCU200 (Ocugen, Malvern, PA, USA)	Bispecific recombinant fusion protein targeting integrin and transferrin receptors	CI-DME	**Phase I Study to Assess the Safety and Efficacy of OCU200 for Center-Involved Diabetic Macular Edema (DME) (DME)**
NCT05940428 (2023–2025)	1	ASKG712 (AskGene Pharma, Camarillo, CA, USA)	Bispecific biologic molecule blocking VEGF and angiopoetin-2 (Ang-2)	DME	**A Study of ASKG712 in Patients With Diabetic Macular Edema** ASKG712 is well tolerated and shows signs of BCVA improvement in preliminary results [282].
NCT05961007 (2021–2024)	1/2	IBE302 (Innovent Biologics, Jiangsu, China)	Bispecific decoy receptor fusion protein targeting VEGF and the complement cascade	DME	**Evaluation of IBI302 Injection in nAMD or DME**
NCT04697758 (2020–2022)	1/2	AXT107 (AsclepiX Therapeutics, Baltimore, MD, USA)	Peptide that inhibits integrin coreceptors of VEGFR [283]	DME	**Safety and Bioactivity of AXT107 in Subjects With Diabetic Macular Edema (CONGO)** The study was terminated as AXT107 resulted in significant adverse effects [284].
**Targeting Novel Pathways**					
NCT04537884 (2020–2022)	1	UBX1325/Foselutoclax (Unity Biotechnology, Foxboro, MA, USA)	Small molecule targeting senescent cells by inhibiting BCL-xL	DME	**Safety and Tolerability Study of UBX1325 in Patients With Diabetic Macular Edema or Neovascular Age-Related Macular Degeneration** **UBX1325 was safe and tolerable, while also showing promise at improving BCVA** [285].
NCT04857996 (2021–2023)	2			CI-DME	**Safety, Tolerability and Evidence of Activity Study of UBX1325 in Patients With Diabetic Macular Edema (BEHOLD)**
NCT06011798 (2023–2025)	3			NPDR and DME	**Assess the Efficacy and Safety of Repeat Intravitreal Injections of Foselutoclax (UBX1325) in Patients With DME (ASPIRE)**
NCT06771271 (2019–2023)	1	RG6179/Vamikibart (Roche, Basel, Switzerland)	Monoclonal antibody inhibiting IL-6	CI-DME	**A Study to Investigate RO7200220 as Monotherapy and in Combination With Ranibizumab in Participants With Diabetic and Uveitic Macular Edema (DOVETAIL)**
NCT05151731 (2021–2025)	2			CI-DME	**A Study to Investigate Vamikibart (RO7200220) in Diabetic Macular Edema**
NCT05151744 (2021–2024)	2			CI-DME	**A Study to Investigate Vamikibart (RO7200220) in Combination With Ranibizumab in Diabetic Macular Edema**
NCT03511898 (2018–2019)	1	THR-149 (Thrombogenics, Oxurion, Leuven, Belgium)	Peptide inhibiting plasma kallikrein	CI-DME	**A Study to Evaluate the Safety of THR-149 in Subjects With Diabetic Macular Edema (DME)** THR-149 was safe and tolerable, with evidence of possible efficacy [286].
NCT04527107 (2020–2023)	1			CI-DME	**A Study to Evaluate THR-149 Treatment for Diabetic Macular Oedema (KALAHARI)**
NCT03666923 (2018–2019)	1	THR-687 (Oxurion, Leuven, Belgium)	Highly selective integrin antagonist	CI-DME	**A Study to Evaluate the Safety of THR-687 in Subjects With Diabetic Macular Edema (DME)** THR-687 was safe and tolerable at all doses, with preliminary efficacy observed in improving BCVA [287].
NCT05063734 (2021–2022)	2			CI-DME	**A Study to Evaluate THR-687 Treatment for Diabetic Macular Oedema. (INTEGRAL)**
NCT06847854 (2022–2027)	1	RO7497372 (Genentech, San Francisco, CA, USA)	---	CI-DME	**A Study to Investigate the Safety, Tolerability, Pharmacokinetics (PK) and Pharmacodynamics (PD) of RO7497372 in Participants With Diabetic Macular Edema (DME) (PREGONDA)**
NCT05699759 (2024)	1	SOM-401	Derivative of a nucleoside reverse transcriptase inhibitor	DME	**Safety and Effect of Intravitreal Injection of a Derivative of Nucleoside Reverse Transcriptase Inhibitor in Subjects with Diabetic Macular Edema**
NCT05919693 (2023–2024)	1/2	MK-3000/Restoret^TM^ (previously EYE-103, EyeBio/Merck, Rahway, NJ, USA)	Tetravalent, tri-specific antibody agonist of the Wnt signalling pathway	DME	**A 2-part Study Consisting of Multiple Ascending Dose (MAD) Safety Study, and a Dose-finding Masked Study to Assess the Safety and Efficacy of Intravitreal (IVT) EYE103 in Patients With Diabetic Macular Edema (DME) or Neovascular Age-related Macular Degeneration (NVAMD) (AMARONE)** Restoret^TM^ was tolerated with no adverse outcomes and observed improves in BCVA and central retinal thickness [288].
NCT06571045 (2024–2027)	2/3			DME	**A Study to Evaluate the Efficacy and Safety of 2 Doses of EYE103 Compared With Ranibizumab (0.5 mg) in Participants With DME (BRUNELLO)**
NCT04424290 (2020–2023)	1/2	BI 764524 (Boehringer Ingelheim, Ingelheim am Rhein, Germany)	Anti-Sema3A antibody reducing areas of ischemia in the retina	DMI	**HORNBILL: A Study to Test Different Doses of BI 764524 in Patients Who Have Had Laser Treatment for a Type of Diabetic Eye Disease Called Diabetic Retinopathy With Diabetic Macular Ischemia (HORNBILL)** BI 764524 was found to be safe and tolerable, and visual outcomes support its further study [289].
NCT06321302 (2024–2026)	2			Moderately severe to severe NPDR	**A Study to Test Whether BI 764524 Helps People With an Eye Condition Called Diabetic Retinopathy (CRIMSON)**

**Table 6:** Implants currently under clinical investigation for the treatment of diabetic retinopathy.

Clinical Trial Identification (Start Year–End Year)	Phase	Drug (Company)	Mechanism of Action	DR Stage	Study Title
NCT04503551 (2020–2025)	3	Susvimo^®^/PDS—Port Delivery System with ranibizumab (Genentech/Roche, San Francisco, CA, USA)	Surgical placed implant at the pars plana that continuously delivers ranibizumab.	CI-DME	A Multicenter, Randomized Study in Participants With Diabetic Retinopathy Without Center-involved Diabetic Macular Edema To Evaluate the Efficacy, Safety, and Pharmacokinetics of Ranibizumab Delivered Via the Port Delivery System Relative to the Comparator Arm
NCT04108156 (2019–2026)	3			CI-DME	A Study to Evaluate Efficacy, Safety & Pharmacokinetics of the Port Delivery System (PDS) With Ranibizumab in Participants With Diabetic Macular Edema (DME) Compared With Intravitreal Ranibizumab; A Substudy to Evaluate the Safety of Re-Implanting the PDS With Ranibizumab in Participants With DME
NCT05695417 (2023–2024)	1	OTX-TKI/Axpaxli^TM^ (Ocular Therapeutix, Bedford, MA, USA)	Intravitreous, bioerodible, hydrogel implant delivering axitinib, an inhibitor of all VEGFRs and PEGFR.	Moderately severe to severe NPDR	Study to Evaluate the Safety, Tolerability, and Efficacy of OTX-TKI in Subjects With Moderately Severe to Severe Non-proliferative Diabetic Retinopathy
NCT03835884 (2019–2022)	1	AR-13503 (Aerie Pharmaceuticals, Durham, NC, USA)	IVT injection of a bioerodible implant release Rho Kinase and PKC inhibitor	DME	A Study Assessing AR-13503 Implant in Subjects With nAMD or DME
NCT06099184 (2024)	2	EYP-1901 (EyePoint Pharmaceuticals, Watertown, MA, USA)	IVT injection of Durasert^®^ technology releasing vorolanib, a small molecule inhibitor of all VEGFRs.	DME	Study of EYP-1901 in Patients With Diabetic Macular Edema (DME) (VERONA)
NCT05383209 (2022–2024)	2			Moderately severe to severe NPDR	Study of EYP-1901 in Patients With Nonproliferative Diabetic Retinopathy (NPDR)
NCT06003751 (2023–2026)	2	PER-001(Perfuse Therapeutics, San Francisco, CA, USA)	Sustained release of a small molecule that inhibits the endothelin pathway	Moderately severe to severe NPDR	A Study of PER-001 in Participants with Diabetic Retinopathy

**Table 7:** Results from the PADOGA trial comparing Suvismo^®^ to monthly injections of ranibizumab [315].

Treatment Arm	PDS Q24W (*n* = 381)	Ranibizumab (*n* = 253)
PDS Q24W	Monthly Injection	Intravitreal injection loading dose every 4 weeks and then implantation of PDS on week 16 with refill-exchange every 24 weeks
Ranibizumab dose	0.5 mg	0.5 mg Q4W then 100 mg/mL
Adjusted mean BCVA change from baseline at 64 weeks	9.4 ETDRS letters	9.6 ETDRS letters
Central subfield thickness at 64 weeks	−203.5 um (95% CI, −212.2 to −194.8)	−199.7 (95% CI, −209.9 to −189.5)
DRSS improvement of ≥2 steps at 64 weeks	39.0% (95%% CI, 34.1% to 43.8%)	41.9% (95% CI, 35.8% to 48.0%)
Adverse events of special interest at 64 weeks	88	34

**Table 8:** Gene therapies currently under clinical investigation for the management of diabetic retinopathy.

Clinical Trial Identification (Start Year–End Year)	Phase	Drug (Company)	Mechanism of Action	Delivery Method	DR Stage	Study Title
**NCT04418427** (2020–2022)	2	ADVM-022 (Adverum Biotechnologies, Redwood City, CA, USA)	AAV expressing aflibercept	Intravitreal Injection	CI-DME	**ADVM-022 Intravitreal Gene Therapy for DME (INFINITY)**
**NCT05930561** (2023–2028)	2	4D-150 (4D Molecular Therapeutics, Emeryville, CA, USA)	R100 vector expressing aflibercept and VEGF-C inhibitory RNAi	Intravitreal Injection	CI-DME	**4D-150 in Patients with Diabetic Macular Edema (SPECTRA)**
**NCT04567550** (2020–2024)	2	ABBV-RGX-314 (REGENXBIO, AbbVie, Rockville, MD, USA)	AAV8 vector expressing a ranbizumab like anti-VEGF monoclonal antibody fragment	Suprachoroidal Injection	NPDR, PDR, or CI-DME	**RGX-314 Gene Therapy Administered in the Suprachoroidal Space for Participants With Diabetic Retinopathy (DR) Without Center Involved-Diabetic Macular Edema (CI-DME) (ALTITUDE)**
**NCT05916391/NCT06492876** (2023–2028)	1/2	FT-003 (Frontera Therapeutics, Watertown, MA, USA)	AAV gene expression system delivering durable expression of intraocular proteins	Subretinal Injection	CI-DME	**A Dose-escalation and Dose-expanded Phase I/II Clinical Study to Evaluate the Safety, and Efficacy of FT-003 in Subjects With DME**
**NCT06237777** (2024–2026)	1	SKG0106 (Skyline Therapeutics, Cambridge, MA, USA)	AAV vector expressing an anti-VEGF, Vb24	Intravitreal Injection	CI-DME	**A Clinical Study Evaluating the Safety, Tolerability and Initial Efficacy of SKG0106 Intravitreal Injection in Diabetic Macular Edema (DME) Patients**

## Data Availability

No new data were created or analyzed in this study.
